# A Review on the Current State and Future Perspectives of [^99m^Tc]Tc-Housed PSMA-i in Prostate Cancer

**DOI:** 10.3390/molecules27092617

**Published:** 2022-04-19

**Authors:** Sara Brunello, Nicola Salvarese, Debora Carpanese, Carolina Gobbi, Laura Melendez-Alafort, Cristina Bolzati

**Affiliations:** 1 Institute of Condensed Matter Chemistry and Technologies for Energy ICMATE-CNR, Corso Stati Uniti 4, 35127 Padova, Italy; sara.brunello@icmate.cnr.it (S.B.); nicola.salvarese@cnr.it (N.S.); 2 Immunology and Molecular Oncology Unit, Veneto Institute of Oncology IOV-IRCCS, Via Gattamelata 64, 35124 Padova, Italy; debora.carpanese@iov.veneto.it; 3 Department of Pharmaceutical and Pharmacological Sciences, University of Padova, Via Marzolo 5, 35131 Padova, Italy; carolina.gobbi@studenti.unipd.it

**Keywords:** technetium-99m, gallium-68, SPECT, PSMA, prostate cancer, target-specific, nanoparticles, molecular imaging

## Abstract

Recently, prostate-specific membrane antigen (PSMA) has gained momentum in tumor nuclear molecular imaging as an excellent target for both the diagnosis and therapy of prostate cancer. Since 2008, after years of preclinical research efforts, a plentitude of radiolabeled compounds mainly based on low molecular weight PSMA inhibitors (PSMA-i) have been described for imaging and theranostic applications, and some of them have been transferred to the clinic. Most of these compounds include radiometals (e.g., ^68^Ga, ^64^Cu, ^177^Lu) for positron emission tomography (PET) imaging or endoradiotherapy. Nowadays, although the development of new PET tracers has caused a significant drop in single-photon emission tomography (SPECT) research programs and the development of new technetium-99m (^99m^Tc) tracers is rare, this radionuclide remains the best atom for SPECT imaging owing to its ideal physical decay properties, convenient availability, and rich and versatile coordination chemistry. Indeed, ^99m^Tc still plays a relevant role in diagnostic nuclear medicine, as the number of clinical examinations based on ^99m^Tc outscores that of PET agents and ^99m^Tc-PSMA SPECT/CT may be a cost-effective alternative for ^68^Ga-PSMA PET/CT. This review aims to give an overview of the specific features of the developed [^99m^Tc]Tc-tagged PSMA agents with particular attention to [^99m^Tc]Tc-PSMA-i. The chemical and pharmacological properties of the latter will be compared and discussed, highlighting the pros and cons with respect to [^68^Ga]Ga-PSMA11.

## 1. Introduction

**Figure 1:**
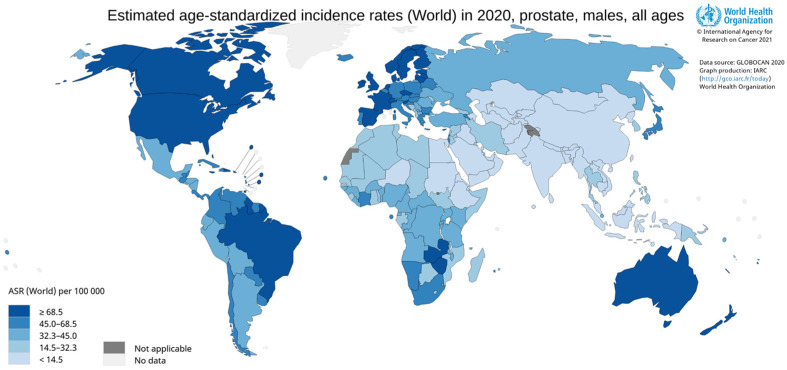
Word incidence rates of prostate cancer in 2020.

Prostate cancer (PCa) is one of the most common malignant cancers in men. In America and Europe, it is the second most common cancer in males, while it is much less frequent in North Africa, eastern Asia, and the Middle East [1,2,3]. In 2020, approximately 1.4 million cases of PCa were detected, and estimates indicate a gradual increase in PCa in the world, with 2.3 million cases predicted in the next 20 years (Figure 1) [4]. The incidence of PCa increases with age and among other risk factors, we can mention race, family history, genetic factors, and lifestyle [5]. PCa occurs in most cases without symptoms and patients present morbid states due to metastases of different severity resulting from these malignancies, which can culminate in death [6].

PCa staging is traditionally based on the sum of Gleason score, prostate-specific antigen (PSA) levels, and clinical stage. For patients with localized PCa, the five-year survival rate is close to 100%, but it dramatically decreased to 30% for patients with distant metastases involving lymph nodes (LN) or/and bones because the treatments available for metastatic PCa have shown weak healing efficacy and recurrence is common after curative treatments. Consequently, an accurate primary staging to determine the extent of PCa is essential for planning treatment in high-risk patients with distant metastases.

Currently, the diagnosis of PCa is mainly based on PSA blood assay [7,8], also used as a screening test, as well as the use of digital rectal examination and transrectal ultrasonography (TRUS)-guided biopsy [9]. Regarding PSA, it presents important limitations in diagnostic accuracy because it is not specific to PCa and can be used as a marker for benign prostatic hyperplasia and prostatic hypertrophy [8,10].

Less invasive imaging modalities, such as magnetic resonance imaging (MRI) and computed tomography (CT), are also employed in the initial evaluation of PCa. These imaging modalities are focused on morphological changes and, therefore, suffer from limited value in the detection of LN metastasis and reduced performance in localizing early bone marrow metastases. Moreover, because of the restricted field of view of MRI in the standard procedure, metastases outside the imaging area are neglected [11]. However, MRI and invasive histopathologic evaluation are standard methods for the detection and staging of primary PCa. Bone scintigraphy has long been utilized for the assessment of bone metastases (BM) with low specificity and sensitivity at low PSA blood levels.

Functional imaging procedures with radionuclides such as single-photon emission tomography (SPECT) and positron emission tomography (PET) offer important advantages over the above-mentioned techniques since they both allow the whole-body detection of functional abnormalities and permit the identification of the multiple molecular and cellular processes that are active in PCa patients. In addition, the availability of multimodal imaging techniques such as SPECT/CT and PET/CT or PET/MRI which combine functional and morphological information allows for high diagnostic accuracy. These hybrid techniques have currently gained a significant role in the clinic as primary staging tools in PCa and in patients with suspected disease recurrence. In addition, they can be helpful to clarify equivocal findings or to detect an additional site of the disease, which could potentially alter patients’ management.

The cornerstone for the successful application of SPECT and PET relies on the overexpression of specific biomarkers in various tumors: receptor proteins, enzymes, and antigens. With regard to PCa, the prostatic-specific membrane antigen (PSMA), discovered by Heroszewicz et al. in 1987 [10,12], continues to increase in importance as a molecular target for the development of target-specific agents for (radio)pharmaceutical applications [10]. Indeed, PSMA has revolutionized the approach to the diagnosis and therapy of PCa. Therapeutic application is possible because of the cytosis-mediated internalization following PSMA ligand binding.

PSMA, also known as N-acetyl-L-aspartyl-L-glutamate (NAAG) peptidase or glutamate carboxypeptidase II (GCPII), is a zinc-containing metal-enzyme transmembrane glycoprotein normally expressed in healthy human tissues and normal prostate epithelium. It is strongly up-regulated in prostate tumor cells and its degree of expression is directly correlated with the stage and grade of tumor progression and malignancy, especially in androgen-independent PCa and metastasized tumor tissues (e.g., LN, bone, lung, rectum) [13,14,15,16,17]. PSMA is also expressed exclusively in the neovasculature of several solid tumors (*vide infra*), being absent in the vasculature of healthy tissues [18]. Due to its selective overexpression, PSMA has been recognized as an ideal target for PCa imaging and therapy.

**Figure 2:**
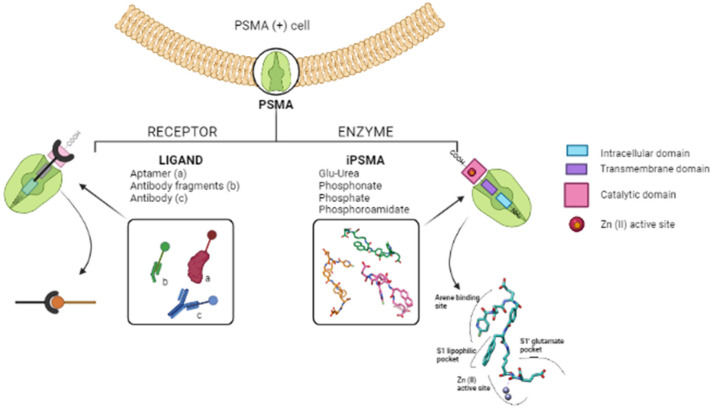
Graphical representation of the dual nature of PSMA biomarker. PSMA consists of three domains: an intracellular domain with 18 amino acids (light blue), a transmembrane region with 24 amino acids (purple), which inserts into the phospholipid bilayer, and a catalytic domain in which there are 707 amino acids (pink) containing the active site with two zinc ions. On the right side, it is possible to appreciate the zoom of the cavity of the active site with a generic PSMA-i ligand in which the portions of the inhibitor that interact with specific entities of the receptor pocket are highlighted (created in BioRender).

Notably, PSMA, owing to its particular localization and structure, can act both as a receptor protein and enzyme (Figure 2) [17,19]. These features have opened the way to two different strategies for targeting with radionuclides [20]. The first is based on the use of large molecules including monoclonal antibodies and derivatives, and aptamers that tightly bind PSMA with high specificity and selectivity [21,22]. In this connection, [^111^In]In-labeled capromab pentapeptide (7E11-C5 antibody), known with the tread-mark of ProstaScint™, was the first PSMA targeting tracer approved by Food Drug Administration in 1997 with the indication for use in PCa patients. ProstaScint™ proved to be efficient, but its clinical use is limited due to different drawbacks that are especially related to its specificity for epitopes of the intracellular domain of PSMA. Consequently, this radiopharmaceutical (RP) is being abandoned [23]. To overcome ProstaScint™ limits, antibodies and derivatives able to bind the extracellular portion of PSMA have been identified and developed (see Section 3.1) [24].

The second exploits the GCPII enzymatic function, which involves the binding of the glutamate-containing structure to the enzymatically active site located in the extracellular domain. This has fostered the design and synthesis of a variety of low-molecular-weight anti-PSMA inhibitors that specifically and selectively interacted with the binuclear zinc active site of the protease. Small inhibitor classes comprise derivatives of phosphonic acid, thiol, and urea [25]. Advantages of the use of such small molecules over antibodies are: easy conjugation of pertinently selected BFCA; easy preparation on a large scale of the construct; favorable pharmacokinetics (fast tumor uptake and rapid excretion).

Among the classes cited above, urea-based derivatives, here referred to as PSMA-i, have been largely exploited for the development of radio-imaging/theranostic probes [17,22,26,27,28,29,30,31,32,33,34].

**Figure 3:**
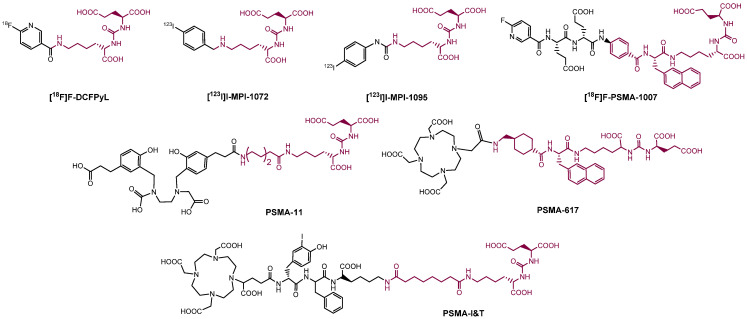
Chemical representation of selected labeled and unlabeled PSMA-i derivatives. PSMA-i pharmacophore is evidenced in color.

Consequently, PSMA-i pharmacophores were conjugated with different functional groups or chelators, characterized by a different degree of lipophilicity, suitable for labeling with radio-halogens (^123,124,131^I and ^18^F) or bivalent and trivalent metal ions (^64^Cu^2+^, ^68^Ga^3+^, ^111^In^3+^, ^177^Lu^3+^ and ^225^Ac^3+^) for tumor molecular imaging and endoradiotherapy [35,36]. The structure of selected PSMA-i agents is reported in Figure 3.

To date, Gallium [^68^Ga]Ga-PSMA-11, is the first and only metal-based RP including a PSMA-i pharmacophore approved by FDA for patients with PCa and suspected biochemical recurrence [37]. It consists of a Glu-Urea-Lys inhibitor motif conjugated via an aminohexanoic (Ahx) spacer to the high Ga-specific acyclic chelator N,N′-bis[2 -hydroxy-5-(carboxyethyl)benzyl]-ethylene-diamine-N,N-diacetic acid (HBED-CC). Clinical trials showed moderate sensitivity (70%) and high specificity (84%) for the detection of local tumor extent and correlation between uptake of the primary tumor Gleason score, PSA levels, and the presence of distant metastases [29,38,39,40,41]. Due to the limited spatial resolution of the instrumentation and the presence of background activity in the urinary tract, some small lesions can be missed. In addition, low sensitivity may be related to low uptake in a tumor with a lower Glison score (<7). [^68^Ga]Ga-PSMA-11 also proved high specificity (95–97%) in LN detection (sensitivity 61–84%), higher sensitivity (97%) and specificity (100%) compared to [^99m^Tc]Tc-bone scan with superior sensitivity compared to MRI, and comparable diagnostic value with respect to Na[^18^F]F PET/CT [29,42,43]. [^68^Ga]Ga-PSMA-11 also outperforms the limited sensitivity and specificity of the [^11^C/^18^F]-choline-based PET/CT agents for the detection of the primary tumor, LN and BM [29].

The good results obtained with [^68^Ga]Ga-PSMA-11 have led to the transposition of its chemistry to γ-emitting radionuclides like ^111^In, and to theranostic radionuclides. Since HBED-CC chelator does not consent the formation of stable chelates with other radiometals, modification of the chelating system was needed, therefore PSMA-I&T [22] and PSMA-617 were developed. As a further example, [^111^In]In-tagged PSMA-I&T has been successfully applied for preoperative SPECT/CT visualization of PCa and as γ-probe for radio-guided resection of PSMA-positive lesions, providing a sensitivity of 83.6%, a specificity of 100% and an accuracy of 93% [44,45].

Despite the advantages listed for both [^68^Ga]Ga-PSMA-11 and [^111^In]In-PSMAI&T there are also some limitations with respect to the physical properties of ^68^Ga and ^111^In radionuclides, which have prompted the development of [^99m^Tc]Tc-PSMA-i agents as a cost-effective alternative to [^68^Ga]Ga- and [^111^In]In-PSMA-i tracers. Additional inputs came from the reduced availability and high cost of the PET technique, which limits its wider spread diffusion all over the world [46,47].

Table 1 summarizes the pros and cons of the use of technetium-99m radionuclide vs. gallium-68 and indium-111 radionuclides.

This review aims to give a concise overview of the specific features of the developed [^99m^Tc]Tc-tagged PSMA agents. A brief description of the currently used Tc-based labeling approaches is reported along with their application for the radiolabeling of both protein derivatives, aptamers, and small PSMA-i. Chemical and pharmacological properties of [^99m^Tc]Tc-PSMA-i will be compared and discussed more in detail, highlighting the pros and cons with respect to [^68^Ga]Ga-PSMA-i tracers, in particular to [^68^Ga][Ga-PSMA-11], as the most frequently employed RP.

## 2. PSMA Expression

**Figure 4:**
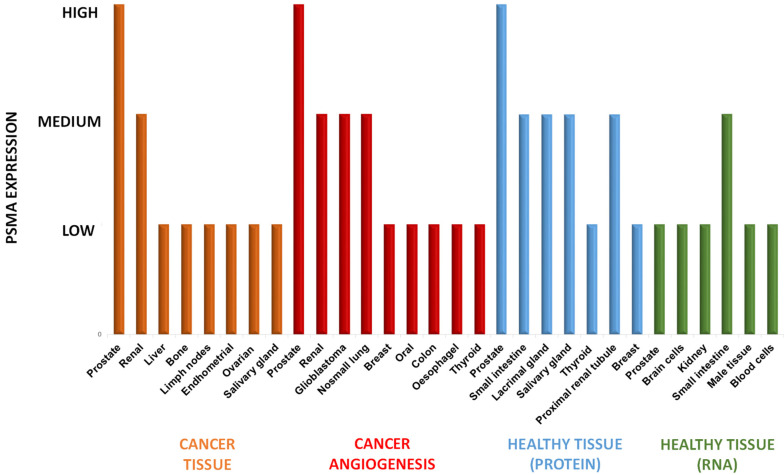
Protein expression of PSMA in cancer tissues, neovascularization of cancer, and healthy tissues, along with the RNA expression in healthy tissues, obtained by combined information from the Human Protein Atlas and data from the literature.

In contradiction to what the name suggests, PSMA is not restricted to PCa cells but it is also expressed in other tumors and neovascular endothelial cells of many tumors, as well as in healthy tissues. Sound knowledge of PSMA distribution may help to avoid being misled concerning the imaging interpretation of radiolabeled-PSMA-i [48]. Figure 4 summarizes the distribution of PSMA in healthy, neoplastic, and neovascular tissues, including the level of RNA expression in healthy tissues obtained by the Human Protein Atlas (Swedish program that maps all human proteins in cells, tissues, and organs using an integration of various omics technologies) combined with the data achieved by literature analysis [19,49,50,51,52,53,54].

Orange bars pointed out the PSMA expression in tumors. The highest levels are encountered for PCa cells (significantly higher than physiological levels). PSMA is upregulated in more than 90% of PCa cells to endorse its use as a biomarker for imaging and treatment of this cancer. Lower degrees of PSMA expression are evident in other malignancies such as renal, liver, bone, lymph nodes, etc. Among these, bone and distant LN are the most common metastatic sites of PCa (frequency 84% and 10.2%, respectively) followed by liver (10.6%).

High or medium-high levels of PSMA are even expressed in the neovascularization of other solid tumors (red bars) [55,56], in particular, in renal carcinoma [50,51], glioblastoma [57,58,59,60,61], non-small cell lung cancer, breast cancer, oral cancer, colon cancer, esophagus cancer and thyroid cancer [19,50,51,52,53,54], meanwhile, PSMA was absent on the membrane surfaces of these tumor cells. Hence, the RP-PSMA-i uptake is mainly related to the expression of the receptor in neovasculature. This evidence has encouraged the use of PSMA as a theranostic biomarker for other solid cancers, although intrapatient tumor heterogeneity has been detected [49].

PSMA is also distributed at medium-high levels in some healthy tissues (light-blue and green bars in Figure 4). This expression implies consideration of possible and potential side effects, in particular, when it is targeted with (radio)therapeutic agents (*vide infra*). However, the expression of the PSMA protein (light-blue bars in Figure 4) is found physiologically in cells of the prostate gland, small intestine in the jejunal brush, proximal renal tubule, thyroid, tubarial glands [62], and breast, but not in the vasculature of healthy tissues [18]. In other healthy tissues, PSMA expression is only at the RNA level (green bars). These are brain cells, such as astrocytes and Schwann cells in the nervous system [63], cells of the secretory acinar epithelium of male tissue, and to a lesser extent, in blood cells [64].

Normal physiological distribution of RP-PSMA-i resembles the baseline PSMA tissues expression as well as non-specific excretion of the agent through renal and hepatobiliary clearance. In general, the most intense radioactivity is appreciated in kidneys with subsequent urinary excretion, as well as in salivary and lacrimal glands, whereas relatively low radioactivity is found in the liver and spleen.

The levels of expression of this enzyme in healthy tissues, especially in kidneys and salivary and lacrimal glands, constitute a worsening of the target/no-target ratio, thus compromising the quality of the diagnostic information; meanwhile, in therapy, important side effects can arise such as dry mouth and eyes, and kidney deficiency. In the case of a high dose of RP, more severe side effects can occur such as xerostomia and hematotoxicity, so the patient cannot proceed with the treatment. However, dry mouth syndrome is the most common shortcoming of PSMA-radiation therapy for prostate cancer. To overcome this and stimulate salivary flow, different strategies have been adopted in patients, even with limited success, these include sialendoscopy with dilatation [65], external cooling of the salivary glands with icepacks, irrigation with saline and steroid injection, as well as intraparenchymal injections of botulin toxin [66]. Of course, a more important limitation involves the kidneys. In this connection, methods to prevent/reduce the radiopharmaceutical uptake levels in this district are under evaluation in animal models [17,67]. They make use of blocking agents for PSMA receptors such as 2-PMPA also developed as orally available prodrugs [68] or monosodium glutamate that injected intraperitoneally (IP) lowered only salivary and renal tubules uptake of [^68^Ga]GaPSMA-11 without affecting the tumor uptake.

## 3. [^99m^Tc]Tc-Labeling Approaches

Over past years, although the development of new PET metal-based agents has led to a contraction in SPECT research programs, and the development of new ^99m^Tc agents becomes quite rare, this radionuclide remains the best one for SPECT imaging, even if most of the currently used agents are more than 20 years old. In addition, the forthcoming introduction into clinical practice of new advanced SPECT cameras based on cadmium zinc telluride (CZT) gamma detectors will significantly reduce differences in sensitivity between PET and SPECT modality, allowing for the collection of images with a much higher spatial resolution that approaches the one-millimeter scale in only a few minutes, outperforming PET imaging [69,70], and paving the way for attractive new clinical opportunities for diagnostic [^99m^Tc]Tc-labeled molecules. In this sense, to fully exploit the potential of these new technologies, novel categories of [^99m^Tc]Tc-compounds are likely to be required, opening a new era for [^99m^Tc]Tc-RPs.

From a radiopharmaceutical standpoint [^99m^Tc]Tc-labeling is still an attractive approach in the preparation of radiolabeled peptides for SPECT imaging [71,72]. Consequently, basic research in ^99m^Tc-RPs has been able to follow trends in molecular imaging, in particular by developing efficient [^99m^Tc]Tc-analogs of [^68^Ga]Ga-RPs [73,74]. Furthermore, the possibility to prepare ^186^Re/^188^Re analogs of the [^99m^Tc][Tc]-labeled tracer can provide important therapeutic strategies, offering the opportunity to develop a tracer for widespread scintigraphic diagnostics with ^99m^Tc and for endoradiotherapy with ^186/188^Re.

**Figure 5:**
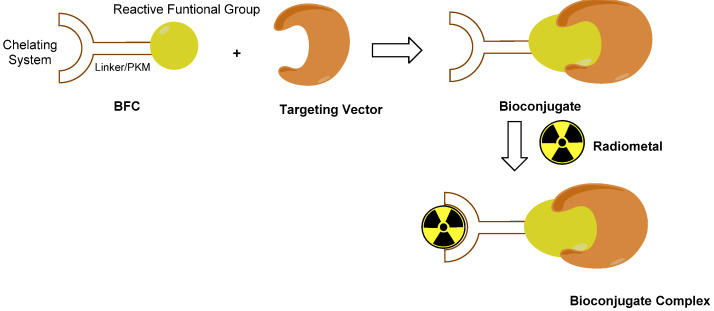
Representation of the biFuntional approach. By this approach, an RP can be described as mainly composed of two essential parts: the targeting vector, i.e., the molecule or macromolecule that drives the radiometal to the pertinent molecular target, and the chelating system (also known as the BiFuntional Chelator, (BFC) designed to promptly and robustly bind the metal radionuclide, preventing it’s in vivo leakage, and to carry another reactive group able to form a strong covalent bond with the targeting vector, thus yielding a kinetically and thermodynamically stable construct. These two parts are directly bound or are held together by an appropriate linker or pharmacokinetic modifier (PKM). The final compound (bioconjugate complex) is then obtained through the appropriate labeling procedure, which depends on the specific radiometal.

The procedure that is commonly used for designing metal-based target-specific RPs is the so-called ‘bifunctional approach’ (Figure 5) [47,71].

During past decades, some techniques based on this bifunctional approach centered on Tc(I) and Tc(V) have been developed and implemented [71,75,76,77,78,79]. These strategies exploit the aptitude of the metal to form characteristic inorganic functional frameworks (also known as ‘cores’, ‘metal-fragments’ or ‘synthons’) with certain organic or inorganic ligands, and the ability of these moieties to tightly bind a single specific donor atom or a small set of specific donor atoms [71]. Accordingly, the donor atom (or the set of donor atoms) is included in the BFC structure, making the coordination to the metal center possible, thus leading to the formation of high thermodynamically and kinetically stable bioconjugated complexes, thus reducing the possibility of in vivo transmetalation by endogenous bi or tri-valent ions.

These approaches are thoroughly discussed in recent reviews [71,75,76,77,78]; Table 2 summarizes the specific features of the main developed methods. Mono-oxo [^99m^Tc][TcO]^3+^ and di-oxo [^99m^Tc][TcO_2_]^+^ technetium cores, Tc-organohydrazine [^99m^Tc][Tc-HYNIC] core and the Tc-tricarbonyl [^99m^Tc][Tc(CO)_3_]^+^-fragment have been widely applied in radiopharmaceutical development to label a number of different molecular vectors [71,75,76,77,78]. In this regard, a leading role has been covered by the Tc-tricarbonyl fragment developed by Alberto and co-workers [75,76,77]. On the contrary, the implementation of [^99m^Tc][TcN(PNP)]^2+^ synthon (PNP = alkoxy-alkyl bisphosphinoamine) is still poorly investigated despite the promising biological performance and very high chemical flexibility shown by the framework [79,80,81,82,83]. Even though none perfectly match the basic requirements for nuclear medicine practice application [84], all of them are suitable for high specific targeting molecules, providing a highly stable product with high specific activity.

Tc-tricarbonyl allows us to obtain well-characterized final products in an easy two-step procedure, which generally requires heating; nevertheless, recent studies indicate that it could be optimized for high RCY at room temperature [85]. Because of their organometallic nature, these compounds tend to be lipophilic. 

Although the mono-oxo [^99m^Tc][TcO]^3+^ allows the efficient preparation of discrete agents by a simple one-step procedure, this system presents important shortcomings that may reduce its application. These include the impossibility to obtain a complex in high specific activity, the difficulty in molecular vector conjugation and backbone derivatization to improve the pharmacokinetics of the bioconjugated complex, and the isomers formation.

Tc-organohydrazine involves a one-step synthesis that is suitable for room temperature; however, the final products are often affected by significant isomerism, the single isomers are difficult to characterize, as well as the chemical structure of the radiocomplexes [78]. Moreover, this approach is hardly achievable for ^188/186^Re, thus an extension to theranostics is not possible [75].

Furthermore, [^99m^Tc][TcN(PNP)]^2+^ leads to the formation of isomers, though they are well established and possess almost the same biological profile [86]; recently, this system was improved by the introduction of a water-soluble PNP ligand, which allows room temperature labeling, and a one-step procedure was truly optimized [83].

All this demonstrates the rich and versatile chemistry of technetium, which is not shared with bivalent or trivalent radiometals. However, no other new and efficient chemical platforms apt for radiolabeling of bioactive molecules have been investigated over the last two decades. Factors such as coordination preferences, stable oxidation states or robust cores of the metal, and ligand selection are fundamental in the design of powerful agents. Therefore, the capacity to introduce new labeling strategies depends on our understanding of both the fundamental coordination chemistry of the metal and of the novelties and advances in the field.

### 3.1. [^99m^Tc]Tc-Labeled Anti-PSMA Antibody and Derivatives

Antibody-based constructs were the first strategy pursued to develop PSMA imaging agents. After approval by the FDA of ProstaScint^®^, with the purpose of overcoming its intrinsic drawbacks, antibodies targeting the extracellular portion of PSMA were studied.

In this connection, Smith-Jones et al. developed the J591an anti-PSMA mAb. It is a humanized mAb that can bind to the extracellular domain of PSMA with high affinity [87]. J591 was pertinently derivatized and radiolabeled with both [^131^I]I- and [^111^In]In. To allow [^111^In]In-labeling, J591 was first conjugated with DOTA (-1,4,7,10-tetraazacyclododecane-N,N,N,N-tetraacetic acid) by direct coupling of one of the four carboxylic acid groups of DOTA to the primary amines in the antibody protein structure. Despite the fact that J591 can target viable tumor cells, its applications as a diagnostic agent are limited as it has a prolonged clearance from soft tissues, like most antibodies, so it is necessary to wait a period of 5–7 days between the injection of J591 for the scan to obtain a good diagnostic image [88]. In spite of this important limitation, this mAb was also radiolabeled with technetium-99m. Nargund et al. were the first to label murine J591 (MUJ591) with ^99m^Tc by a direct method using photoreduction of disulphide bonds to generate free thiols for metal coordination, to stage early prostate cancer before radical surgery [88]. The authors found that there was no correlation between radiopharmaceutical uptake and PSA levels or histological grading and that [^99m^Tc]Tc-MUJ591 scintigraphy was not useful for obtaining information on tumor volume. Therefore, it was concluded that [^99m^Tc]Tc-MUJ591 could be useful to localize the recurrence of the disease after radical prostatectomy, but it was not sensitive enough to delineate microinvasion of the capsule, seminal vesicles or bladder neck [88].

*Engineered Ab fragments*. It is common knowledge that the most critical point to consider when labeling a large molecule such as mAb is the physical half-life of the radionuclide, which has to match with the biological half-life of the biomolecules to provide synchronous activity. This is important to ensure that there is sufficient time for the radioimmunoconjugate to accumulate in the tumor site before radionuclide decay, to allow for good tumor visualization, and that radiation exposure to normal tissues is as low as possible. Intact mAb have long in vivo half-lives (weeks/days) that are responsible for slow pharmacokinetics, low target-to-non-target ratios, and elimination via the hepatobiliary system [89]. These features do not match the physical properties of technetium-99m and other long-living radionuclides such as zirconium-89 and lutetium-177 are more useful for this purpose.

Antibodies can be engineered into smaller fragments that largely retain the original antigen-binding properties but with the advantage of a more favorable clearance and kidney elimination, optimal tumor-to-non-tumor ratios in shorter times (8 h *p.i.*), to allow good sensitivity and contrast imaging on the day of injection. This permits the usage of medium-lived radionuclides such as copper-64 or relatively short-lived radionuclides such as technetium-99m, with the benefit of an appreciable reduction in absorbed dose by patients [89].

Hence, to improve the pharmacokinetic profile of anti-PSMA mAbs-engineered antibody fragments such as diaboby (dimeric bivalent form of scFv, ~50 kDa) were developed. Owing to its intermediate size, diabody allows for a good balance between circulation time/systemic clearance, target accumulation, and tissue penetration compared to the corresponding whole protein. In preclinical studies, good contrast images can be obtained within 1 to 8 h *p.i.* [89]. Kampmeier et al. synthesized a [^99m^Tc]Tc-tagged diabody derived from mAb J591 and evaluated its potential as a SPECT imaging agent in mice bearing DU145-PMSA xenografts tumors [90]. J591 diaboby bound to PSMA(+) cells with low nanomolar affinity (3.3 ± 0.2 nM), it was site-specifically labeled with ^99m^Tc-tricarbonyl via the C-terminal His-tag. animal studies showed slow blood clearance and late tumor visualization (4 h after injection), with the best contrast 8 h after injection [90].

The engineering of single-chain variable region fragments (scFv; MW 27 kDa) of Ab is another way to improve their pharmacokinetics for imaging with short half-lived radionuclides. Parker et al. developed a J591(scFv) based on the reported complementarity determining region of J591 [91]. Later, Nawaz et al. were the first to prepare J591-scFv incorporating a His-tag for the labeling with [^99m^Tc][Tc(CO)_3_]^+^-fragment [92]. [^99m^Tc]Tc-J591scFv was obtained, after 60 min of heating at 37 °C, with radiochemical yields ranging from 85 to 90%. Upon the purification by gel filtration, the final radiochemical purity was 99% and the maximum specific activity achieved was 7 MBq/μg. In vitro studies demonstrated that the purified complex was stable in serum and showed selective binding to PSMA-positive cells [92]. In a very recent work, authors optimized the His-tags sequence incorporated into the J591scFv, to enhance the radiochemical yields of [^99m^Tc][Tc(CO)_3_]^+^-J591-scFv under mild conditions of pH and temperature, thus avoiding post-labeling purification [93]. Six consecutive His residues in the protein sequence, surrounded by several positively charged residues (Arg or Lys), and the presence of phosphate in the buffer are required for optimal radiolabeling. Actually, under these conditions, an scFv targeted to the PSMA antigen can be readily labeled in >95% radiochemical yield, without the need for subsequent purification [93]. The collected in vivo studies clearly evidenced that the scFv fragment has better characteristics than diabodies for the detection of PSMA-positive tumors [90,92].

D2B murine mAb is one of the best anti-PSMA antibodies reported in the literature, so far. It showed promising results in PCa studies efficiently targeting PSMA and inducing internalization [94]. The scFv-D2B derivative has shown a high binding affinity to the PSMA receptor and optimal internalization capability of the complex (40%). These features were maintained after being radiolabeled with different long-lived radionuclides (^111^In; ^177^Lu). Remarkably, studies of the radiolabeled [^177^Lu]Lu-scFv-D2B clearly showed good pharmacokinetics and greater affinity for PSMA in vitro and in vivo than the radiolabeled Glu-ureido-based PSMA inhibitors [95,96], making it a promising candidate for PCa early diagnosis [94].

*New strategies.* Gene therapy using aptamers is another approach against prostate cancer to convey siRNA that suppresses the overexpression of the oncogene MDM2 associated with PCA. In particular, it is possible to exploit the chimera siRNA-Apt that, thanks to an appropriate bonded chelating agent, is able to chelate the ^99m^Tc [97,98,99,100]. The bifunctional chelating agent succinimidyl 6-hydraziniumnicotinate (SHNH) combined with synergistic binders EDDA and Tricine, as well as ^99m^TcO_4_^−^ under the action of a reducing agent SnCl_2,_ has been chosen to solve the labeling problems and low stability of nucleic acids. The advantages of this method are that the aptamers can be easily labeled with good RCY producing complexes that are chemically stable [98].

### 3.2. [^99m^Tc]Tc-Housed PSMA-i

Over past years, various [^99m^Tc][Tc]-PSMA agents were developed. Structural variations involving the nature of the chelator, the length of the spacer between the BFCA and the PSMA-i scaffold, as well as the presence of an additional carboxyl group either close to the chelator or on the linker backbone have been considered (vide infra).

**Figure 6:**
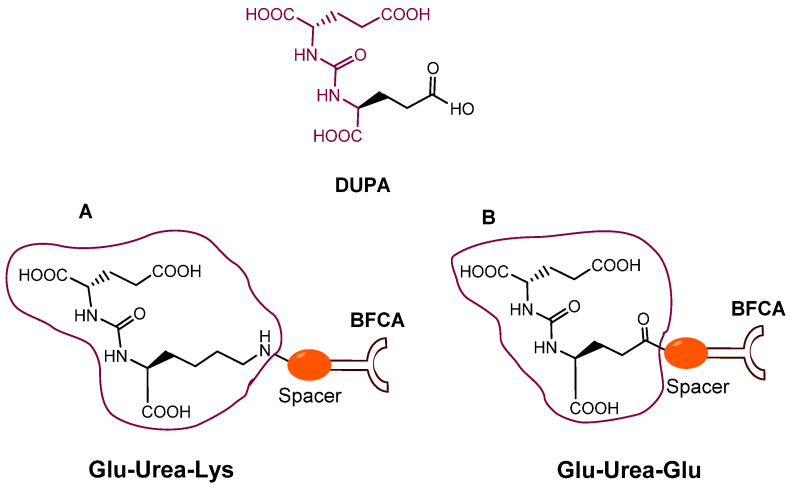
Chemical sketches of DUPA and PSMA-i. (**A**,**B**) Glu-Ureo-based PSMA inhibitors conjugated through a spacer (in orange) to a so-called Bifunctional Chelating Agent (BFCA).

Since 2007, a number of different [^99m^Tc]Tc-radiolabeled compounds, mainly based on low-molecular-weight PSMA inhibitors, have been explored for imaging applications. Some of them were based on the phosphoroamidate motif or thiol to target PSMA [101,102,103]; meanwhile, the most promising were built on the glutamate-urea pharmacophore (**Glu-Urea**-Glu or **Glu-Urea**-Lys) as derivatives of 2-[3-(1,3-dicarboxypropyl)ureido]pentanedioic acid (DUPA), able to bind PSMA-positive cells with nanomolar affinity (Figure 6). The glutamate portion of these frameworks binds the S1′pharmacophore pocket and the ureido functional group efficiently coordinates the Zn^2+^ ions of the enzymatic active site of PSMA (*vide infra*) [28].

**Figure 7:**
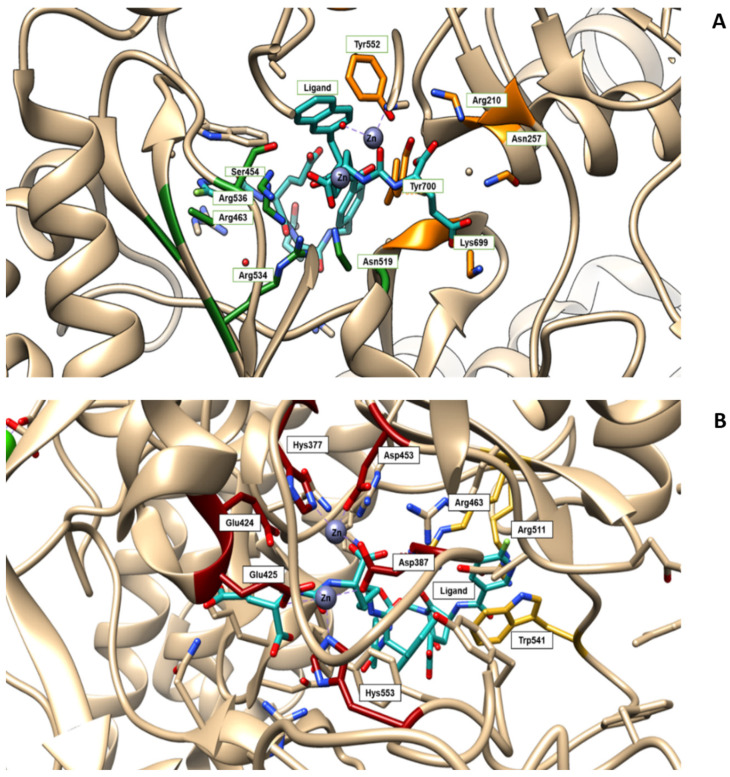
(**A**) Amino acids of the active site of the PSMA enzyme (GCPII) interacting with the ligand PSMA 1007 (PDB code 5O5T, in light-blue). S1 pocket (‘’non-pharmacophore pocket’’) with its arginine patch is indicated in green. It is specific for binding to the NAA (N-acetyl-aspartyl-) portion of NAAG (or the NAA-like- portion of PSMA-i) through polar or nonpolar interaction [112]. In orange is highlighted the S1′ pocket, which specifically binds C-terminal glutamate residue. S1 is a flexible funnel specific for negatively charged amino acids which improve the interaction between PSMA and their inhibitors; its flexibility enables the binding of a variety of groups, which are not essential to the determination of the affinity [113]. (**B**) The active site of the PSMA receptor in which amino acids deputy to stabilize zinc ions are colored in red, whereas the side chains of Arg463, Arg511 and Trp54, forming the “arene-binding site”, are colored in yellow. The latter define the entrance of GCPII [114]. The images are created using UCSF Chimera 1.14.

As a general rule, according to the bifunctional approach, the development of these agents requires the attachment of a relatively *bulky* BFCA to the Glu-Urea- pharmacophore to accommodate the radiometal (Figure 6). Furthermore, as for gallium agents, the insertion of an appropriate spacer separating the Glu-Urea portion from the chelator is essential to direct the [^99m^Tc]Tc-chelate through the 20 Å tunnel away from the active site of PSMA, thus facilitating the housing of the pharmacophore group into the enzymatic binding site or to improve the binding potential and pharmacokinetics of the tracer [27,104]. To give an example, Figure 7 shows the specific interaction of PSMA1007 with the active site.

In this connection, outcomes from different investigations clearly showed that small molecule inhibitors exploiting the Glu-Urea-Glu motif possess a PSMA affinity higher than that of the corresponding Glu-Urea-Lys counterpart. Additionally, the presence of a linker between the pharmacophore and the chelating moiety is mandatory to obtain high binding affinity, while linkers containing at least seven methylene units between the chelator nitrogen and the amide carbonyl (e.g., Ahx) give complexes of low nanomolar affinity. Moreover, the inclusion of aromatic substituents (e.g., D-Try- D-Phe; D-Try-D-2-Nal; L-Trp) on the spacer back bone has a positive effect on both binding affinity and pharmacokinetics of the radioconjugate. At the binding pocket, they enhance the interaction of the linker unit with the remote arene-binding site of the PSMA receptor Figure 7).

From the pharmacokinetic point of view, the presence of aromatic substituents supports the binding of radiolabeled compounds to plasma protein to delay its blood clearance and achieve maximum internalization of the tracer in tumor cells, and increase uptake at tumor sites [34,105,106,107,108,109,110,111].

**Figure 8:**
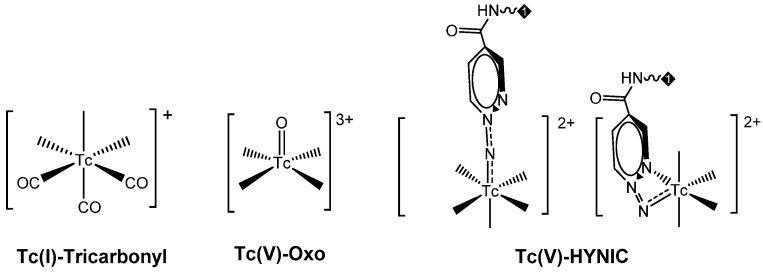
Chemical representation of the Tc-moieties utilized in developing [^99m^Tc]Tc-housed PSMA-i.

PSMA11 and PSMA617, respectively, based on (Ahx)-Lys-Urea-Glu and (Nal)-Lys-Urea-Glu structural motifs (Figure 6), are the most commonly used scaffolds for theranostic application. In PSMA-617, the two-linker moieties 2-naphthyl-L-alanine and 4-(aminomethyl)cyclohexanecarboxylic acid have shown a positive impact on RP pharmacokinetics, leading to a high internalization ratio and consequently high image contrast. They were also conjugated with chelators for radiolabeling with technetium tricarbonyl [^99m^Tc][Tc(CO)_3_]^+^-fragment, and the technetium oxo [^99m^Tc][TcO]^3+^ and technetium organohydrazine [^99m^Tc][Tc-HYNIC]^2+^ cores (Figure 8), thus giving a considerable number of agents (Table 3, Table 4, Table 5 and Table 6).

Each system has a specific chemical demand arising from its fundamental characteristics, which results in distinct preferences for the ligand donor atoms, the coordination number, and geometry [71]. Indeed, the selection of appropriate chelators or coligand systems allows for the formation of highly thermodynamically and kinetically stable complexes as well as for a fine modulation of the radioconjugate binding affinity and pharmacokinetics by adjustment of their overall chemical-physical properties: overall charge, polarity, and hydrophobicity (vide infra).

Although several [^99m^Tc][Tc]-tagged PSMA inhibitors have been developed and evaluated in preclinical studies for PCa detection, results from these investigations have disclosed that most of these agents possess some drawbacks concerning radiosyntheses and full chemical characterizations (unclear structures) of the radioconstructs, as well as the lack of uniformity in the expression of receptor affinity data that make a straight comparison between the different [^99m^Tc][Tc]-PSMA-i difficult.

With regard to radiosyntheses, in general, efficient and reliable kit procedures have been developed using the different labeling approaches, even if some issues have to be pointed out. These rely on the BFCA-linker-PSMA-i chemical properties, which are responsible for the relatively low labeling efficiency. Actually, the usage of organic solvents (e.g., alcohols, acetonitrile, DMSO, NH_4_Ac 0.5 M pH 8) is necessary to improve the solubility of PSMA-i ligands, as well as the use of amounts of ligands in the range 10^−4^–10^−5^ M for each preparation vial. Heating at 90–100 °C for an extended time (30–60 min) is also required to produce the final radiocompound in acceptable radiochemical yield (70–95%). In particular, with the exception of [^99m^Tc][Tc-HYNIC]-tagged PSMA-is that are produced with high radiochemical yield and purity (>98%) through a lyophilized vial kit, the preparation of [^99m^Tc][Tc(CO)_3_]- and [^99m^Tc][TcO]-labeled PSMA-i required an additional purification step (by HPLC or solid-phase extraction cartridge) to obtain the agent with a degree of radiochemical purity > 95%, suitable upon dilution with PBS for biological evaluations. For [^99m^Tc][TcO]-labeled PSMA-i, the presence of α-D-glucoheptonate or disodium tartrate as exchanging ligands is necessary to stabilize the [^99m^Tc][TcO]-core in a pre-reduced intermediate, thus reducing the formation of [^99m^Tc][TcO_2_]. In addition, despite [^99m^Tc][Tc-HYNIC]-tagged PSMA-i being efficiently produced, their chemical characterization is difficult to ascertain and this could make the approval of the new drugs difficult.

The affinity of [^99m^Tc]Tc-housed PSMA-i agents was generally assessed in vitro using LNCaP and different subclones of PC-3 cell lines. The former is a human PCa cell line that naturally expresses PSMA at a high level. PC-3 cells are PSMA negative human androgen-independent PCa cells derived from BMs. They were genetically engineered to stably overexpress PSMA (PC3-PIP) or transduced with the flu peptide (PC-3flu) as a control cell line. The literature describes different methods to determine the affinity of the molecules for PSMA making a direct comparison rather difficult. Hence, receptor affinity was determined by measuring the relative binding affinity of the non-radiolabeled ligands and the corresponding ^Nat^Re-complexes using the N-acetylated-α-linked acidic dipeptidase (NAALADase) assay in LNCaP cell lysate extracts and expressing the data as K_i_ values (enzyme inhibition constant; nM) generated by Cheng-Prusoff conversion [105,106]. Instead, the affinity expressed as the IC_50_ (nM) value of the ligand precursor and ^Nat^Re-complexes was determined by a competitive binding assay using LNCaP cells/cell membranes and a radioligand such as [^125^I](I-BA)KuE ((S)-1-carboxy-5-(4-(-^125^I-iodo-benzamido)pentyl)carbamoyl)-l-glutamic acid) as a standard [115]. ^131^I–MIP1095 ((S)-2-(3-((S)-1-carboxy-5-(3-(4[ I]iodophenyl)ureido)pentyl)ureido)pentanedioic acid) was also utilized [111]. Again, affinity as K_D_ (dissociation constant, nM) was achieved by the saturation binding test using an increasing concentration of ^99m^Tc-complex on LNCaP cells in the presence or in the absence of an excess of competitor (2-phosphonomethyl pentanedioic acid, 2-PMPA) [116]. The binding specificity of the radioconstructs was determined by cell affinity and internalization studies in PSMA-positive and negative cell lines in the presence or absence of an excess of 2-PMPA [116,117]. The data of all investigated species are summarized in Table 3, Table 4, Table 5 and Table 6.

**Table 4:** PSMA-i labeled with [^99m^Tc][Tc(CO)_3_]^+^-fragment in a multivalent approach.

	Chelator/Linker/Scaffold	Affinity	Performance	Ref.
**[Tc(CO)_3_]15–16** **(2020)**	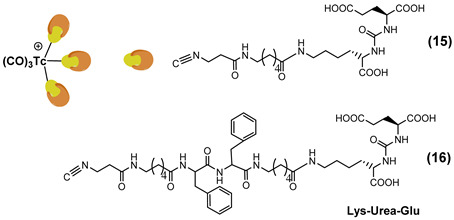	^a^ 5.50 ± 0.99 (15) 0.20 ± 0.01 (16)	Complexes were investigated on 22Rv1 xenografts. Moderate PSMA-dependent tumor uptake 1.87 ± 0.11% ID/g, at 1 h *p.i*, which increased to 2.83 ± 0.26% ID/g at 4 h. **Clinical translation:** No	[118]
	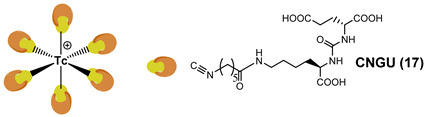	^b^ 8.79 (17)	Complexes were investigated on LNCaP xenografts. PSMA mediated tumor uptake: 4.86 ± 1.19% ID/g at 1 h *p.i*. High kidneys 70.95 ± 12.28% ID/g and spleen 5.84 ± 1.51% ID/g uptakes. Tumor-to-blood ratio was 2.89 and tumor–to–muscle ratio was 12.46. Liver and intestinal uptake were 2.46 ± 0.72 and 2.16 ± 0.34% ID/g, respectively. **Clinical translation:** No	[119]

^a^ PSMA inhibitory affinity was determined using the corresponding technetium-99m complexes. Data are reported as K_D_ (nM). 22Rv1 are PSMA-positive human prostate carcinoma epithelial cells, which have moderate PSMA expression [19,120]. ^b^ The binding affinity to PSMA of the unlabeled CNGU ligand was performed by using NAALADase assay on LNCaP cell lysates. Data are reported as the enzyme inhibitory constant (*K*i nM).

**Table 5:** PSMA-i labeled with [^99m^Tc][TcO]^3+^-core.

	Chelator/Linker	Scaffold	Affinity	Performance	Ref.
**[TcO]** **1–6** **(2009)**	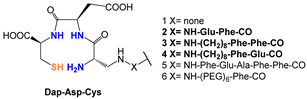	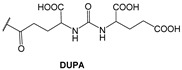	^a^ 176 (1) 60 (2) 13.8 (3) 31 (4) 102 (5) 338 (6)	Complexes were evaluated on LNCaP xenografts. High and stable PSMA-dependent tumor uptake (11.2% ID/g at 4 h *p.i.*). High kidney uptake 28.9% ID/g. Low non-target organs uptake (**<1**% ID/g at 4 h *p.i.*) High target-to-non target ratios **Clinical translation:** No	[116]
**[TcO]** **7–14** **(2013)**	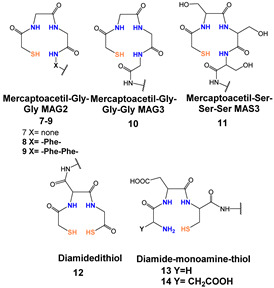	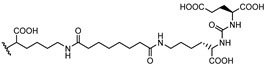	**^b^** 0.03 (7) 0.13 (8) 1.91 (9) 7.99 (10) 0.18 (11) 0.05 (12) 0.17 (13) 2.73 (14)	Complexes were evaluated on PC3-PIP/PC3-flu xenografts. **[TcO]7,11,14** are the best of the series. High and stable PSMA-dependent tumor uptake (26.81 ± 1.9–33.59 ± 3.20% ID/g at 2 h *p.i.*). High kidney uptake followed by slow washout. High spleen uptake followed by slow elimination Low non-target organ uptake (**<2**% ID/g at 1 h *p.i.*) High target-to-non target ratios **Clinical translation:** No	[106]
**[TcO]** **15,16** **(2017)**	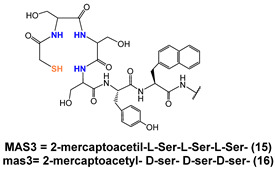	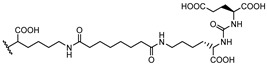	^c^ 47.6 ± 2.5 (15) 39.7 ± 1.2 (16)	[TcO]**16** was evaluated on LNCaP xenografts. High PSMA-dependent tumor uptake (8.28 ± 3.27% ID/g at 1 h *p.i.*). High kidney uptake and spleen uptake. Low non-target organs uptake (**<3**% ID/g at 1 h *p.i.*). **Clinical translation:** Yes, Phase 2	[115]

^a^ PSMA inhibitory affinity was determined by using the technetium-99m complexes. Data are reported as K_D_ (nM); ^b^ PSMA inhibitory affinity was determined by using the ligands, and data are reported as K_i_ (nM). ^c^ PSMA inhibitory affinity was determined by using the corresponding ^Nat^Re complexes, data are reported as IC_50_ (nM).

**Table 6:** PSMA-i labeled with [^99m^Tc][Tc-HYNIC]^2+^-core.

	HYNIC-PSMA-i Scaffold	Affinity	Performance Clinical Translation	Ref.
**[Tc-HYNIC]1** **(2013)**	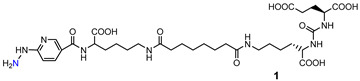	^a^ 1.74	Complexes were evaluated on PC3-PIP/PC3-flu xenografts. Whole-body SPECT-CT imaging show low PSMA-dependent tumor uptake and high background activity. **Clinical translation:** No	[106]
**[Tc-HYNIC]2** **(2017)**	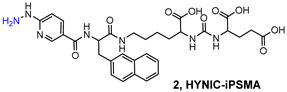	n.d	Complex was evaluated on LNCaP/PC3 xenografts. High and stable PSMA-dependent tumor uptake (9.84 ± 2.63%I D/g at 3 h *p.i.*). High kidney uptake followed by slow washout (12.63 ± 0.56%ID/g at 3 h *p.i.*). Low nontarget organs uptake (**<2**% ID/g at 1 h *p.i.*). High target-to-nontarget ratios: Tumor-to-blood 271 and tumor-to-muscle 246 at 3 h *p.i.* **Clinical translation:** Yes, phase 1	[117]
**[Tc-HYNIC]3** **(2017)**	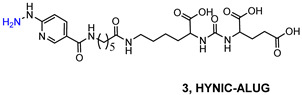	**n.d**	Complex was evaluated on LNCaP/PC3 xenografts. High and stable PSMA-dependent tumor uptake (19.45 ± 2.14% ID/g at 2 h *p.i*). High kidney uptake followed by slow washout. Low nontarget organs uptake (**<3**% ID/g at 1 h *p.i.*) High target-to-non-target ratios: Tumor-to-blood 24.33 at 23 h *p.i.* **Clinical translation:** Yes, phase 1	[109]
**[Tc-HYNIC]4-6** **(2020)**	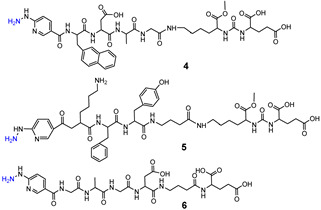	^b^ 13.58 23.63 42.36	Complexes were evaluated on LNCaP/PC3 xenografts. PSMA-dependent tumor uptake (3.62 ± 0.78–1.8 ± 0.32% ID/g at 2 h *p.i.*). High kidney uptake. Low nontarget organs uptake. **Clinical translation:** No	[110]
**[Tc-HYNIC]7** **(2020)**	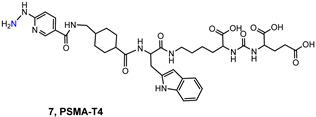	^c^ 80 (7)	Complex was evaluated in healthy BALB/C mice. No data on LNCaP/PC3 xenografts were reported. Reduced kidney uptake (37.5 ± 9.5) at 4 h *p.i.* with respect to other ^99m^Tc-PSMA. Low nontarget organs uptake (**<1**%ID/g at 4 h *p.i*). **Clinical translation:** Yes, phase 1	[111]

^a^ PSMA inhibitory affinity was determined using the ligand, data are reported as K_i_ (nM); ^b^ PSMA inhibitory affinity was determined by using the corresponding technetium-99m complexes. Data are reported as K_D_ (nM). ^c^ PSMA inhibitory affinity was determined by using the ligand precursor in competition studies, data are reported as IC_50_ (nM).

In vivo specificity has been evaluated using PSMA(+)/PSMA(−) xenograft tumor-bearing mouse models. All evaluated compounds had PSMA-dependent tumor uptake, which was validated by blocking studies with the 2-PMPA inhibitor. Again, the pharmacokinetics was derived from the different chemical-physical features of [^99m^Tc]Tc-housed PSMA-i agents including charge, shape, and lipophilicity. The in vivo performance of each complex is summarized in Table 3, Table 4, Table 5 and Table 6.

*[^99m^Tc][Tc(CO)_3_]-Labeled PSMA-i.* [^99m^Tc][Tc(CO)_3_]^+^-fragment was generally complexed using the single amino acid chelate (SAAC) concept, based on the lysine residue (Table 3), which embraces a tridentate chelating entity with different degrees of steric hindrance and lipophilicity, and a terminal reactive group to conjugate directly, or with the interposition of a linker to the PSMA-i scaffold by solid-phase synthesis (SPPS). This approach generated a series of differently charged agents whose structures and features are summarized in Table 3 [34,105,106,107,108].

The John Hopkins group, in a pioneering work, described a series of Lys-Urea-Glu-based agents (Table 3) [105,106]. The Lys free ε-amino group of the PSMA-i was conjugated directly or through the insertion of a linker to different chelators, which were selected to generate radioconstructs with different chemical-physical properties. However, as all compounds possessed the same Lys-Urea-Glu motif, structural variation derived from the length of the linker, the chelator (bis-pyridyl, bis-quinoline, or mixed mono-pyridyl mono-carboxyl), the presence or absence of a second amide function between BFCA and the first amides attached to the lysine moiety, and the presence or absence of a carboxyl group either adjacent to the chelator or adjacent to the second (linker) amide group [105,106]. Radiosyntheses were performed in two steps using the Isolink kit at ligand concertation of 10^−4^M. RCY 60–70%. Upon complexation to [^99m^Tc][Tc(CO)_3_], fragment ligands 1 and 2 provided monocationic complexes, whereas 4 was a neutral one. Compounds were stable in vitro and in vivo. In vitro binding studies clearly show the impact of the linker length on the PSMA affinity of the constructs. Thus, linkers containing at least seven methylene units between the chelator nitrogen and the amide carbonyl give complexes of low nanomolar affinity, in the absence of the liker ([^99m^Tc][Tc(CO)_3_]**6**) a significant reduction in the inhibitory activity was observed. Imaging studies were performed on all radioconstructs by using SCID mice bearing both PSMA(+) PC3-PIP and PSMA(−) PC3-flu xenografts. With the exclusion of [^99m^Tc][Tc(CO)_3_]**6,7**, compounds enabled the visualization of the PC3-PIP tumor and kidney, both expressing PSMA. The distribution was dependent on the whole physical-chemical features of the complexes. Biodistribution studies were performed on [^99m^Tc][Tc(CO)_3_]**1,3** selected as the lead compounds. Data showed for the tracers a PSMA-dependent tumor uptake in PC3-PIP xenografts. For both tracers, the tumor uptake was highest at 30 min post-injection (*p.i.*) (7.87 ± 3.95 for [^99m^Tc][Tc(CO)_3_]**1** and 11.56 ± 2.86 for [^99m^Tc][Tc(CO)_3_]**3**) but was followed by washout (~0.75–0.80% at 5 h *p.i.*); a similar trend was also observed in spleen and kidney, meanwhile, the distributions within normal organs/tissues were favorable.

Taking advantage of the above results, in a subsequent paper, the same group assessed three additional [^99m^Tc][Tc(CO)_3_]-tagged Lys-Urea-Glu-based agents [106]. Among the tested compounds the neutral [^99m^Tc][Tc(CO)_3_]**8** showed superior pharmacokinetics and promising SPECT-CT images compared to the other agents considered (Table 3). Furthermore, it showed very high specific and persistent uptake in PSMA(+) PC3-PIP tumors. Renal uptake was also high at early times *p.i.*, but was followed by slow washout (122.70 ± 14.73 and 55.31 ± 1.15% IA/g at 0.5 and 5 h *p.i.*), as well as the spleen uptake (21.11 ± 8.49 and 1.83 ± 1.52% IA/g at 0.5 and 5 h *p.i.*). Despite its promising distribution profile and good target-to-nontarget ratio which are consistent with the clinical translation, no clinical studies with this compound are reported.

**Figure 9:**
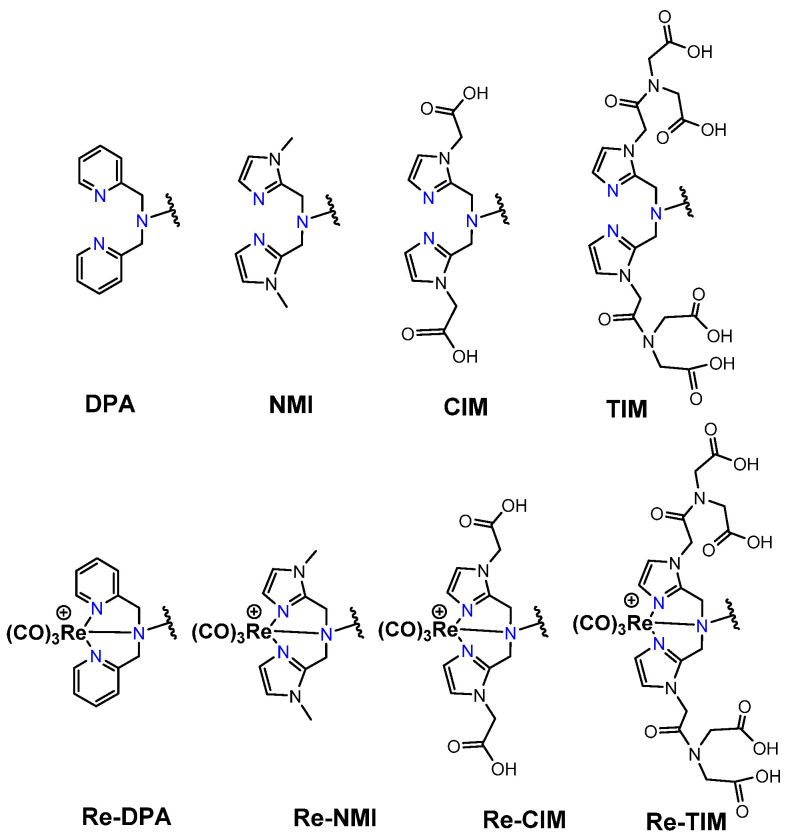
General structure of the SAAC Lys-based bifunctional chelator and corresponding Re complexes.

Another series of [^99m^Tc][Tc(CO)_3_]-based PSMA-i complexes has been reported by Babich et al. at Molecular Insight Pharmaceuticals. Inc. [34,107]. The compounds were designed on Lys-Urea-Glu and Glu-Urea-Glu scaffolds, which were coupled through various linkers to four different SAAC ligands (Figure 9): bis(pyridin-2-ylmethyl)amino (DPA), bis((1-methyl-1H-imidazol-2-yl)methyl)amino (NMI), bis((1-(carboxymethyl) -1H-imidazol-2-yl)methyl)amino (CIM) or bis(1-(2-(bis(carboxymethyl)amino)-2- oxoethyl)-1H-imidazol-2-yl)methyl)amino (TIM) [34]. The impact of the chelate (metal = Rhenium), as well as the length and composition of the linker on the in vitro affinity, was firstly evaluated in human prostate cancer LNCaP cells [34].

The use of polar CIM and TIM chelators (**11**–**14** in Table 3), which contain additional carboxylic groups, afforded metal constructs exhibiting a three or four-fold improvement in the binding affinity for PSMA with respect to the other compounds. The direct attachment of the SAAC ligand to the Glu-Urea-motif led to inactive species, suggesting that the presence of a linker between the pharmacophore and the chelating moiety was mandatory to obtain a higher binding affinity. Meanwhile, the length of the spacer does not show a strong influence on the binding properties; only a moderate increase in the affinity was detected, expanding the length of the spacer from 3 to 11 units. The use of Lys as a linker reduced the affinity of Lys-Urea-Glu-based agents. Replacement of the Lys amino acid with a Glu residue (Glu-Urea-Glu scaffold) significantly improved the inhibitory activity of the compounds that was 4-5 times higher than the analogs. These efforts led to the identification of a diverse series of promising PSMA inhibitors (Table 3), which were further investigated in animal models of human PCa [107]. CIM and TIM are based on two imidazole derivatives and contain three nitrogen atoms for the coordination of the [^99m^Tc(I)(CO)_3_]^+^ moiety to yield monocationic compounds; they differ in the pharmacokinetic modifying groups attached to the chelator backbone. Each imidazole in MIP-1405 (**11**) and MPI-1427 (**13**) has one terminal carboxymethyl group, while MPI-1428 (**12**) and MIP-1404 (**14**) have a bis-carboxymethyl amino-2-oxoethyl group attached to each heterocycle. The *tert*-butyl ester-protected precursor molecules were used for radiolabeling in preliminary studies. Radiosyntheses were performed in two steps using the Isolink™ kit to generate the reactive [^99m^Tc][Tc(CO)_3_(H_2_O)_3_] intermediate, which after neutralization was reacted at 100 °C for 30 min with a ligand concentration of 10^−4^ M in equal volume mixture of water and acetonitrile. RCY was 70%. Before biological studies, purification by HPLC was necessary. However, a single vial kit was realized to prepare [^99m^Tc][Tc(CO)_3_-MIP-1404 and selected as a lead compound of the series [108]. It was achieved by heating the fully deprotected MIP-1404 ligand (100 μg) at 100 °C for 60 min with the neutralized [^99m^Tc][Tc(CO)_3_(H_2_O)_3_]^+^. Upon complexation, the estimated RCY was >85% and RCP > 92% on a 200 mCi scale (specific activity of >2000 mCi/μmol). High-affinity, saturable binding to PSMA on LNCaP cells was observed for the four investigated compounds. The uptake was highly specific, as demonstrated by blocking studies. Radioconstructs were shown to internalize at 37 °C. In LNCaP xenografts, the complexes showed a high and persistent tumor uptake (Table 3) and high tumor-to-blood ratios at 4 h *p.i*. [107]. [^99m^Tc][Tc(CO)_3_-MIP-1404 exhibited the best combination of high tumor uptake and rapid clearance from kidney and nontarget tissues as proved by SPECT/CT imaging. It is currently in phase III clinical trials.

Like carbonyl (C=O) functional groups, isonitrile (C≡N-R) groups are reported to be excellent ligands for stabilizing technetium in low oxidation states. Actually, they can strongly coordinate the [^99m^Tc][Tc(CO)_3_]^+^ fragment in a monodentate fashion, producing monocationic, six-coordinated complexes of the type [^99m^Tc][Tc(CO)_3_(CN-R)_3_] [121]. By combing this set of coordinating atoms and taking advantage of the intrinsic coordination properties of Tc(I) ion, a [^99m^Tc][Tc(CO)_3_]-PSMA-i probe possessing multivalent targeting moieties has recently been proposed by Lodhi et al. [118]. Isonitrile ligands were pertinently derived to carry a Lys-Urea-Glu-based scaffold that permits, in principle, the simultaneous binding of three PSMA receptor sites (Table 4).

[^99m^Tc]Tc-**15** and [^99m^Tc]Tc-**16** were obtained at high radiochemical efficiency (≥98.5%) adopting a two-step synthesis. Upon purification, RCP was ≥99.5% and the molar activity of the products was 4.07 × 10^8^ GBq/mol. Both compounds are stable and disclosed a high and specific PSMA binding affinity in PSMA-expressing 22Rv1 cells (human prostate carcinoma epithelial cells, which have moderate PSMA expression [19,120]. The K_D_ value of [^99m^Tc]Tc-**16** was comparable to that of [^99m^Tc]Tc-MIP-1404. Animal studies were carried out in 22Rv1 tumor xenografts over four hours. [^99m^Tc]Tc-**16** was the best performing agent, showing a rapid but moderate accumulation at the tumor site (Table 4). The uptake was receptor-mediated. The feasibility of [^99m^Tc]Tc-**16** as a PSMA imaging agent was tested by SPECT/CT using the same animal model. [^99m^Tc]Tc-**16** clearly allows for the visualization of the tumor 1 h post-injection and showed prolonged retention of activity until 4 h. A complex was cleared from non-targeted tissues and showed improved images with high tumor-to-background contrast at 4 h post-injection. Despite promising results, this work suffers from the absence of a comparative study with other previously reported molecules conducted under exactly the same experimental conditions. Thus, it is difficult establishing whether this approach has improved with respect to the monovalent approach.

Isonitrile groups can also coordinate with Tc(I) ion to form very stable monocationic six-coordinated homoleptic compounds. Therefore, very recently, Xiao et al. using C≡N-R as a bifunctional monodentate ligand prepared a [^99m^Tc][Tc^I^(CN-R)_6_]^+^ complex and evaluated it for PCa imaging [119]. The ligand (20 µg for each preparation; final volume 350 µL) was radiolabeled with ^99m^Tc in a simple way and the [^99m^Tc]Tc-CNGU was obtained without further purification with a high RCP (97.3 ± 0.06%) after 20 min of incubation at 100 °C. The specific activity was approximately 8.22 ± 10^5^ GBq/mol. The result of the biodistribution study of [^99m^Tc]Tc-CNGU in LNCaP tumor-bearing mice is summarized in Table 4. The uptake in the tumor of [^99m^Tc]Tc-CNGU was specific to PSMA. The kidney, as a PSMA-expressing and excretory organ, exhibited the highest uptake at 1 h post-injection, as well as the spleen. In the blocking study, a significant reduction in tumor, kidney and spleen uptakes was observed suggesting that the process is receptor-mediated. Uptake by the liver and intestine was low, demonstrating that it was excreted through the renal system rather than the hepatobiliary route. The low uptake in the thyroid and stomach indicated that the complex was stable in vivo.

*[^99m^Tc][TcO]-labeled PSMA-i:* In complexes with the [^99m^Tc][TcO]^3+^ moiety, the core was stabilized by σ-and π-donating atoms, such as amino, amido and thiolate. In this context, tetradentate ligands of the N_4−x_S_x_ class have been investigated to produce monoanionic or neutral complexes [113,122,123], with the advantage of neither requiring additional co-ligands to complete the metal coordination sphere as for the [^99m^Tc][Tc-HYNIC]-approach. The assembly of PSMA-i-linker-BFCA was, in general, accomplished by standard fluorenylmethoxycarbonyl (Fmoc) SPPS. The ligands used in this approach are sketched in Table 5 along with the PSMA inhibitory affinity values and the performance of the resulting [^99m^Tc][TcO]- PSMA-i complexes.

By exploiting the ability of the diaminopropionic acid (Dap)-Asp-Cys chelator to bound and stabilize the [^99m^Tc][TcO]^3+^core Kularatne and coworkers described a series of PSMA-targeting agents for PCa imaging (Table 5, [^99m^Tc][TcO]**1–6**) [116]. In this report, six different PSMA-targeting ligands were designed and selected using silicon docking studies. All include the DUPA framework linked to the Dap-Asp-Cys chelator via spacers of varying lengths and chemistry. Radiosyntheses were performed efficiently using a single vial kit with a ligand amount in the range of 0.065–0.125 mg for the kit. No purification was necessary before in vitro and in vivo treatment. Among the tested compounds, three DUPA radioconjugates [^99m^Tc][TcO]**2**–**4**, with 7 to 16 atom spacers, were found to target PSMA with nanomolar affinity and high specificity, also displaying high and persistent tumor uptake in vivo (average 9.8 ± 2.4% ID/g at 4 h *p.i.*) and little or no-accumulation in other tissues except the kidneys. Meanwhile, DUPA radio-conjugates with longer or shorter spacers exhibited much weaker affinity and lower specificity for the target. Outcomes from this study revealed that the conjugation of the Glu-Urea scaffold to the 8-aminooctanoic acid (to avoid steric overlap within the narrow regions at the base of the tunnel) followed by two phenylalanine residues (for maximum interaction with hydrophobic pockets near the mouth of the tunnel) may allow the production of radioconstructs with high affinity and specificity and with good potential for clinical translation.

In a more comprehensive investigation aimed at exploring the effect of different chelators on the biological profile of 12 PSMA radioconjugates, Benarjee and co-workers described another series of [^99m^Tc][TcO]-tagged PSMA-i (Table 5) [106]. Traditional N_x_S_y_-based chelators with varying charge and polarity were conjugated to Glu-Urea-Lys-(Ahx) scaffold through different linker moieties, thus investigating the effect of aromatic substituents of the spacer on pharmacokinetics (**7**–**14**). MAG_2_, MAG_3_, MAS_3_ and diamidedithiol chelators were selected to produce mononegative [^99m^Tc][TcO]complexes, the diamide-monoamide–thiol to give neutral species. Radiolabeling of **7**–**14** was accomplished by incubating the ligands (concentration 10^−5^ M) with sodium pertechnetate at 90–100 °C for 20 min (RCY = 70–90%). Upon HPLC purification, the radioconstructs were obtained with an RCP ≥ 98% and a specific activity > 411 GBq/µmol. By excluding **12**, the other ligands were used in the S-trityl protected form to facilitate UV detection and HPLC purification. [^99m^Tc][TcO]**7–9** and [^99m^Tc][TcO]**12** containing MAG_2_ and diamide-dithiole chelator were obtained as *syn* and *anti* diastereomers, due to the relative orientation of the functional group with respect to the mono-oxo core, while for the MAG_3_ and MAS_3_ radioconjugated only a single peak was detected during the labeling. All compounds showed a high binding affinity to PSMA irrespective of their charge and lipophilicity, and for [^99m^Tc][TcO]**7–9,12** irrespective of the isomeric form. Biodistribution studies showed a PSMA-dependent binding in PSMA(+) PC3-PIP/PC3-flu tumor xenografts for the tracers. The tumor uptake was rapid, high, and persistent (~20% at 5 h *p.i.*); the distribution within normal organs/tissues (except kidney) was also favorable. The highest nonspecific uptake was noticed in the liver and spleen at 30 min *p.i.*, however, the value decreased over time. Differences in the pharmacokinetic profiles and in kidney uptake of these series of compounds were observed as a result of the different charge, polarity, and presence of a diverse number of hydrophilic functional groups on the chelator backbone. The existence of carboxylic groups on the chelator side chain ([^99m^Tc][TcO]**11** vs. [^99m^Tc][TcO]**14**) allows for a higher tumor uptake and less accumulation of radioactivity in normal tissues, including the spleen, resulting in higher target-to-non target ratios [106].

With the main purpose of developing a [^99m^Tc]Tc-tagged probe for PSMA-targeted radio-guided surgery, a [^99m^Tc]Tc-analog of ^111^In-PSMA-I&T ([^111^In]In-DOTAGA-(3-iodo-y)-f-k-Sub(KuE)) where PSMA is prostate-specific membrane antigen and I&T is imaging and therapy) has been described by Robu et al. [115]. Thus, to meet the clinical need for a more cost-effective alternative to^111^In-PSMA-I&T, the PSMA-I&T scaffold was adapted to the requirement of Tc-chemistry by replacing the DOTAGA chelator with MAS_3_ (Table 5). The 3-iodo- D-Try-D-Phe sequence of the linker was replaced by D-Try-D-2-Nal to enhance the interaction of the linker unit with the remote arene binding site of the PSMA receptor and to support plasma protein binding of the [^99m^Tc]Tc-agent in order to delay blood clearance, thus achieving maximal tracer internalization in tumor cells and increasing the uptake in the metastatic nodes. Moreover, to investigate the vulnerability of all-L-aminoacid MAS_3_ chelator to endopeptidase action, the corresponding mas_3_ (2-mercaptoacetyl- D-ser- D-ser-D-ser-) analog was evaluated in parallel. Solid-phase peptide strategy combined with solution-phase chemistry was developed to prepare the PSMA-construct. The standard labeling protocol was applied to produce the radioconstructs [106,116], C-18 cartridge purification was necessary to remove a substantial amount of ^99m^TcO_2_. However, a lyophilized kit was also settled, exploiting the initial reaction condition. To the freeze-dried vial containing 25 nmol of peptide, sodium hydrogen phosphate buffer, sodium tartrate buffer, and 40 µg of stannous chloride dehydrate, 1-5 mL ^99m^Tc-pertechnetate in saline were added. The kit was heated to 90 °C for 20 min to give the final radioconstruct in a quantitative amount. The final pH of the reaction mixture was 7. Specific activity 44-52 GBq/µmol [115]. No detectable influence of the chelator stereochemistry on the quantitative formation of [^99m^Tc][TcO]-MAS_3_/mas_3_ complexes was observed that was independent of the spatial orientation of the serine side chain. Marked differences in the internalization efficiencies of [^99m^Tc][TcO]-MAS_3_- and [^99m^Tc][TcO]-mas_3_-derivatives were observed. [^99m^Tc][TcO]-mas_3_ showed enhanced internalization in LNCaP cells when compared to its counterpart and values that reached those obtained for ^111^In-PSMA-I&T. Data from in vivo metabolism of the two tracers disclose a lower stability of [^99m^Tc][TcO]-MAS_3_ compared to [^99m^Tc][TcO]-mas_3_ for which the sole intact compound was found in blood, kidney, and urine at 1 h *p.i*.

Because of its improved internalization efficiency and in vivo stability, [^99m^Tc][TcO]-mas_3_- PSMA-I&T (^99m^Tc-PSMA-I&T) was selected for further in vivo investigation. In preclinical studies, compared with ^111^In-PSMA-I&T, ^99m^Tc-PSMA-I&T showed delayed clearance kinetics but identical uptake in PSMA(+) tissues in the LNCaP xenograft model at 1 h after injection (Table 5). The uptake was highly specific, as demonstrated by blocking studies. On the basis of these promising results, ^99m^Tc-PSMA-I&T was introduced and evaluated as RadioGuided Surgery (RGS) imaging probe in patients with PCa (Phase 2).

*[^99m^Tc][TcHYNIC]-labeled PSMA-i.* In spite of the intrinsic chemical drawbacks of this approach, hydrazinonicotinamide (HINYC) is a routine [^99m^Tc]Tc-chelating agent in radiopharmaceutical applications.

HYNIC (6-hydrazinopyridine-3-carboxylic acid) is considered an efficient bifunctional coupling agent for [^99m^Tc]Tc-labeling of large and small bioactive molecules. Here, the reactive carboxyl group was exploited as the anchor site to conjugate the PSMA-i pharmacophore (Table 6), while the hydrazinopyridine portion strongly binds the metal forming a robust core, the diazenido unit (M=N=N-R), easily accessible from the monooxo [^99m^Tc][TcO]^3+^ core by a simple condensation reaction [106,109,110,111,117]. Ancillary ligands are usually required to stabilize the Tc-HYNIC core; hence, EDDA (ethylenediamine-N, N″-diacetic acid) and tricine were always included in the mixture of reagents required for the preparation of the RP [122]. The use of this approach reduces the lipophilicity of radiolabeled preparations, especially those containing the [^99m^Tc][Tc(CO)_3_]^+^-system. However, on the other side, PSMA agents containing HYNIC as BFCA exhibited a high accumulation in the kidneys, which can cause difficulties in the interpretation of scintigraphic images. Banerjee et al. first investigated the application of this approach in an extensive report in which the effect of different chelators for technetium on the binding of PSMA was compared [106].

In 2017, Ferro-Flores and co-workers described the preparation and the clinical translation of [^99m^Tc]Tc-EDDA/HYNIC-PSMA [117]. HYNIC-PSMA bifunctional ligand incorporates the –Lys(Nal)-Urea-Glu inhibitor motif as the pharmacophore, conjugated to a HYNIC group for metal coordination (Table 6). [^99m^Tc]Tc-EDDA/HYNIC-PSMA was quantitatively obtained from a lyophilized cold kit (RCY > 99%) and utilized without the need for post-labeling purification. The specific activity was 14GBq/µmol. In vitro and in vivo studies showed high radiopharmaceutical stability of the radioconstruct and specific recognition for PSMA receptor binding and internalization in cells. Preclinical studies in LNCaP bearing animal models revealed a high and almost stable tumor uptake (Table 6), with little accumulation in other non-target organs, excluding the kidney and liver. Blood clearance was rapid, and washout occurred mainly through the kidneys and liver. By virtue of these encouraging results, [^99m^Tc]Tc-EDDA/HYNIC-PSMA was evaluated in humans [117] (see Section 4, Clinical Investigation).

Another reported example of a bifunctional ligand derived from HYNIC is HYNIC-ALUG (ALUG = aminocaproic acid-Lys-Urea-Glu) [109]. This derivative (see Table 6) was efficiently radiolabeled by applying the routine procedure, which required tricine and EDDA ancillary ligands. Preliminary experiments in PSMA(+) LNCaP cells demonstrated the binding affinity of [^99m^Tc]Tc-HYNIC-ALUG for PSMA. This observation was supported by small-animal imaging in SCID mice bearing LNCaP tumor xenografts showing high uptake of the tracer at 2 h *p.i*. Conversely, no uptake was observed in tumors grown from PSMA-negative PC3 cells, and a marked reduction in LNCaP tumor accumulation was detected in blocking studies, indicating that the uptake is a receptor-mediated process. The kidneys were the primary excretory organs; a small amount of radioactivity was distributed in the intestine, demonstrating that it is partially excreted through the hepatobiliary route. These results were confirmed by ex vivo biodistribution experiments. Actually, [^99m^Tc]Tc-HYNIC-ALUG showed a very high radioactive uptake at tumor sites (19.45 ± 2.14% IA/g at 2 h *p.i.*) which was maintained at 4 h *p.i.* (11.23 ± 2.8% IA/g, and optimal tumor-to-blood ratio (24.23 ± 3.54 at 2 h *p.i.*). Although this agent possesses a high and constant tumor uptake absolutely comparable to that of other reported [^99m^Tc]Tc-PSMA-i inhibitors, it’s high uptake and long retention in the kidney constitute an important shortcoming that needs to be addressed.

Mosayebnia et al. designed and assessed via an in silico docking study a set of HYNIC-peptides bearing the well-known Lys-Urea-Glu scaffold (**3** [Glu-Urea-Lys(OMe)-Gly-Ala-Asp-(Naphthyl)Ala-HYNIC], and **4** [Glu-Urea-Lys (OMe)-GABA-Tyr-Phe-Lys-HYNIC]) and a new non-urea containing pharmacophore (**5** Glu-GABA-Asp-Gly-Ala-Gly-HYNIC) [110]. The chemical structure of the three inhibitors is sketched in Table 6 along with their properties. Molecular docking studies of the three peptides clearly showed that Peptide **3** was found to bind the PSMA active site with high affinity, it showed high binding affinity and formed several interactions with important residues of the receptor. Peptide **5** failed to form some interactions, in particular the bound of the urea moiety with Gly518 and zinc ion. Peptide **4** lacked most of the important interactions with the active site and exhibited the lowest binding affinity. The peptides were synthesized and efficiently radiolabeled. [^99m^Tc][Tc]HYNIC-**3** exhibited the highest affinity for PSMA, probably due to the presence of 2-Naphthylalanine (2-NA) residue in the spacer which interacts with its hydrophobic pocket. In addition, Glu-GABA-Asp was introduced as a new pharmacophore-targeting PSMA that manifested good binding properties in vitro. Data from animal studies reveal good stability and great capability for [^99m^Tc]TcHYNIC-**3** in the visualization of tumor regions after 60 min, as shown by SPECT-CT imaging. Moreover, by considering the pharmacokinetic and binding properties, it was roughly comparable to [^68^Ga][Ga]-PSMA-11. Thus, [^99m^Tc]TcHYNIC-**3** seems to be applicable as a promising SPECT imaging [110].

Recently, investigators from the National Center for Nuclear Research Radioisotopes Centre POLATOM exploiting the HYNIC approach developed a new radioligand, referred to as [^99m^Tc][Tc]-PSMAT4 (PSMA-T4 = HYNIC-4Amc-LTrp-Lys-Urea-Glu), useful in the diagnosis of PCa in patients [111]. The chemical structure of this new tracer along with some information obtained by the preclinical investigation is reported in Table 6. [^99m^Tc][Tc]-PSMAT4 was prepared from a dry kit, which contained 23 mg of PSMA-T4 ligand. Preparation was carried out by adding 1–2.5 mL of sodium pertechnetate to the reaction vial and heating for 20 min at 100 °C. Biodistribution was performed on healthy mice. Data indicate that at 4 *p.i.*, the compound presents a low accumulation in nontarget-organs, in particular, a relatively low kidney uptake was observed (Table 6). It was evaluated in clinical studies, such as in whole-body PSMA tumor detection and biochemical recurrence of PCa.

### 3.3. [^99m^Tc]Tc-Labeled Nanoparticle Carrying PSMA-i

Multimodal properties of nanoparticles (NPs), such as simultaneously carrying drugs and/or diagnostic probes for site-specific delivery, make them excellent tools for the diagnosis and treatment of prostate cancer [123]. Nano-technology actually can be of evaluable help in personalized medicine, as it is based on molecular diagnosis, and prescription of the most appropriate therapeutic treatment, distributed to the target in the most effective mode [124]. Over the years, different attempts to develop [^99m^Tc]Tc-tagged NPs have been reported and the use of radiolabeled NPs was digested in some exhaustive reviews [124,125].

Several inorganic and organic NPs pertinently coated with targeting vectors (e.g., RGD, bombesin, etc.) were efficiently tagged with ^99m^Tc according to the BFC approach with [^99m^Tc][Tc(CO)_3_]^+^-fragment, [^99m^Tc][TcO]^3+^ and [^99m^Tc][Tc-HYNIC]^2+^ cores. However, to our knowledge, only one example of [^99m^Tc]Tc-NP-PSMA-i is described in the literature [126].

**Figure 10:**
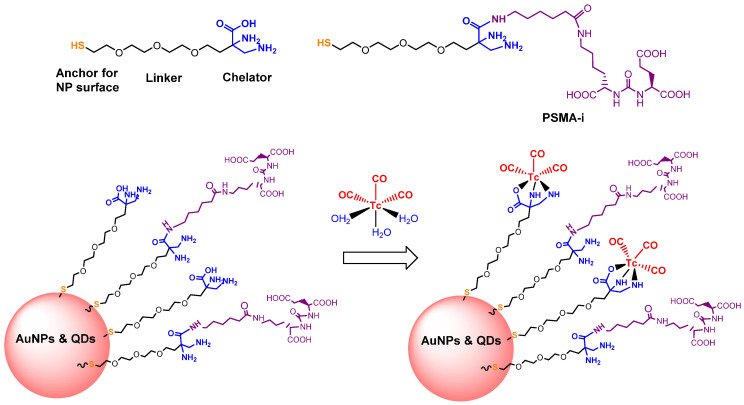
Coating ligands and radiolabeled PSMA-i derivatized NPs designed by Felber and coworkers.

In this work, Felber and coworkers synthesized a new multifunctional coating ligand for gold nanoparticles (AuNPs) and CdSe/ZnS core-shell quantum dots (QDs). The ligand, named HS-PEG-DAP, was formulated with a thiol group as an anchor for the NP surface, a polyethylene glycol (PEG) linker and the 2,3-diaminopropionic acid (DAP) framework that can be utilized as a chelator for [^99m^Tc][Tc(CO)_3_]^+^-fragment or modified to carry functional groups for covalent binding to any biological vector (Figure 10). A small PSMA-i was also conjugated to the coating ligand as a targeting function for PCa.

Derivatized NPs were efficiently labeled with [^99m^Tc][Tc(CO)_3_]^+^- in PBS. For AuNPs, the best RCY was achieved with a temperature gradient from 50 to 70 °C over 1 h and a subsequent incubation for another hour at 70 °C. Whereas, for QDs the labeling temperature should not exceed 60 °C to avoid the formation of precipitates. The in vitro stability and cellular uptake were examined in LNCaP cells. AuNPs, in particular, were highly stable; moreover, cell studies showed active uptake of AuNPs indicative of an interaction between the targeting function of the AuNPs and the PSMA enzyme. Thus, imaging with small animal microSPECT and ex vivo biodistribution studies in LNCaP xenografts nude NMRI mice was carried out. Unfortunately, imaging studies showed a rapid clearance by the spleen and liver with a short time of circulation in the blood that is not sufficient for the NPs accumulation in the tumor [126].

## 4. Clinical Investigations

In addition to the requirements of an efficient labeling procedure and a full chemical characterization, further considerations for clinical translation include: high concentration within the tumor (% ID/g); high binding specificity determined by the PSMA^+^-to-PSMA^−^ cell uptake ratio; longer retention in PSMA^+^ tumor site versus the kidney; and low liver and gastrointestinal uptake. Among the above-mentioned [^99m^Tc]Tc-tagged peptides as PSMA inhibitors for PCa SPECT imaging, five have entered clinical trials, where safety and tolerability were evaluated, as well as the determination of key variables such as sensitivity and specificity.

**Figure 11:**
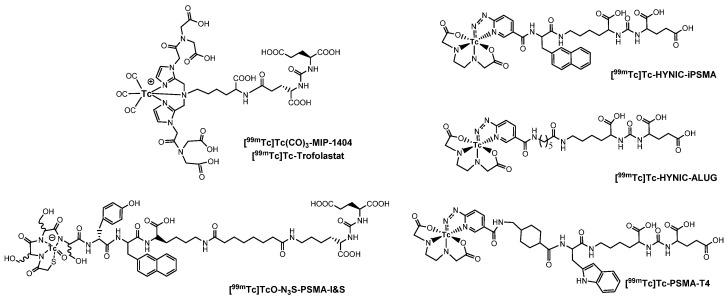
Putative structures of [^99m^Tc]Tc-tagged-PSMA-i under clinical investigation.

The chemical structures of these agents are depicted in Figure 11.

With the exclusion of [^99m^Tc][Tc(CO)_3_]-MIP-1404 ([^99m^Tc]Tc-Trofolastat) all shared the Glu-Urea-Lys moiety. [^99m^Tc]Tc-Trofolastat is the only technetium-99m-based RP developed for PCa SPECT imaging to achieve and complete Phase 3 in the clinical trial (https://clinicaltrials.gov) [127]. It is a six-coordinated monocationic compound in which the *fac*[^99m^Tc][Tc(CO)_3_]-fragment is bound to a BFCA formed by the bis((1-(2-(bis(carboxymethyl)amino)-2-oxoethyl)-1H-imidazol-2-yl)methyl)amino (TIM) chelator attached to a Glu-Urea-Glu pharmacophore [34,107]. The second ^99m^Tc-tagged PSMA agent is the ^99m^Tc-PSMA-I&S (for Imaging and Surgery) [115]. It is composed of a monooxo-[TcO]^3+^ core coordinated to a mercaptoacetyl-tri-D-serine chelator through the terminal thiol sulfur atom and the three nitrogen atoms. The mas_3_ chelator, thanks to the presence of D-aminoacid residues, is stable to the endopeptidase action. Currently, ^99m^Tc-PSMA-I&S is under Phase 2 clinical trials (ClinicalTrials.gov Identifier: NCT04832958). Other [^99m^Tc]Tc-PSMA RPs under clinic investigation are ^99m^Tc-EDDA/HYNIC-Lys(Nal)Urea-Glu (^99m^Tc-EDDA/HYNIC-PSMA) [117], ^99m^Tc-HYNIC-ALUG [109] and ^99m^Tc-PSMA-T4 [111] that used HYNIC as BFCA agent. They have lately been assessed to improve the diagnosis and monitoring of therapy of PCa patients [109,117,128].

Table 7 summarizes the preparation of each [^99m^Tc]Tc-PSMA RPs along with the indication and the advantages and disadvantages.

The first RP for SPECT imaging was developed at Molecular Insight Pharmaceuticals (MIP) and labeled with ^123^I or ^99m^Tc [107]. Vallabhajosula and colleagues performed the first studies using a [^99m^Tc]Tc-tagged PSMA-i compound in healthy men and patients with metastatic PCa, to compare the biodistribution, pharmacokinetics, tumor uptake, and radiation dosimetry of two pioneering agents, the [^99m^Tc]Tc-MIP-1404 and the [^99m^Tc]Tc-MIP-1405 (see Table 3) [129]. The authors demonstrated that both agents were rapidly cleared from the bloodstream, showing acceptable uptake in the liver and kidney, as well as a high and persistent concentration in the lacrimal and salivary glands in healthy men. SPECT/CT images of both agents were useful for rapid localization of bone, LPs, and prostate lesions in patients with PCa. However, [^99m^Tc]Tc-MIP-1404 showed less urinary excretion, and consequently less bladder activity than [^99m^Tc]Tc-1405 [129]. Therefore, it was selected for the phase II prospective study in patients with intermediate and high-grade PCa [130]. Subsequently, Rathke et al. compared [^99m^Tc]Tc-MIP-1404 ([^99m^Tc]Tc-Trofolastat) with [^99m^Tc]Tc-MDP in conventional bone scanning to assess the rate of detection of BM. The study highlighted how the PSMA agent reduced the number of equivocal findings in most patients since several suspect lesions were detected with PSMA scanning compared with bone scanning [131]. The authors explained that they did not include [^68^Ga]Ga-PSMA agents in the comparison because the advantage of the PET over the SPECT imaging modality was predictable thanks to the higher inherent resolution and the better signal-to-noise ratio of the PET technique. Schmidkonz et al. reported that [^99m^Tc]Tc-MIP-1404 allows the detection of LN or BM by SPECT/CT imaging visual evaluation with moderate accuracy at primary staging and minimal interobserver variability. The results demonstrated that total uptake values in primary prostate tumors could predict LN and BM with a sensitivity of 82% and a specificity of 76% [5]. The authors stated that the confidence of visual evaluation can be increased using quantitative SPECT/CT imaging, which is highly reproducible and shows better agreement among observers [132]. The total uptake of [^99m^Tc]Tc-MIP-1404 in the primary tumor was also found to correlate significantly with the Glison Score and PSA serum concentration [5,133,134,135].

Reinfelder et al. performed a visual evaluation of the SPECT/CT scan after [^99m^Tc]Tc-1404 administration in 115 patients and found that for prostate-specific antigen (PSA) levels greater than 2 ng/mL, the detection rate (91.4%) and lesion contrast values were comparable to the data obtained with [^68^Ga]Ga-PSMA PET/CT imaging (74.2–83.8%) in three previously reported studies. However, when PSA levels are below 2 ng/mL the detection rate of [^99m^Tc]Tc-MIP-1404 (40.0%) was lower than that of [^68^Ga]Ga-PSMA-11 (68.8%) [134]. Similar results were reported by Schmidkonz et al. in 225 patients with biochemical recurrence of PCa [133]. The authors found high detection rates (90%) of [^99m^Tc]Tc-MIP-1404 SPECT/CT for PSA levels higher than 2 ng/mL, comparable to those of [^68^Ga]Ga-PSMA-11 PET/CT, but for PSA levels below 1.0 ng/mL the detection rates decrease to 58%. Consequently, it was concluded that [^99m^Tc]Tc-MIP-1404 SPECT/CT imaging is more likely to detect PSMA-positive lesions in patients with elevated levels of PSA [133]. Nevertheless, Schmidkonz et al. have recently demonstrated the high performance of [^99m^Tc]Tc-MIP-1404 also for the detection of PSMA-positive lesions in patients who presented biochemical recurrence of PCa with low (from 0.5 to 1 ng/mL) and very low (0.2 and 0.5 ng/mL) serum PSA levels. The authors reported that the detection rates at low serum PSA levels (56%) were slightly lower than those obtained for [^68^Ga]Ga-PSMA-11 PET/CT (58 and 74%); however, this result was not unexpected in view of the significantly higher spatial resolution offered by PET over SPECT [136].

[^99m^Tc]Tc-MIP-1404 has also been used to evaluate the response to treatment in patients with metastatic PCa [5,132] and with biochemical recurrence of PCa [5] who undergo androgen deprivation therapy or external beam radiotherapy. The agreement between the biochemical response to treatment and SPECT/CT imaging suggests that [^99m^Tc]Tc-MIP-1404 could be a promising agent for the evaluation and monitoring of PCa treatment [5,133].

Based on the positive outcomes of Phase 1 and 2 clinical trials, a Phase 3 study was carried out to evaluate both the specificity of [^99m^Tc]Tc-MIP-1404 image assessment to correctly identify patients without clinically significant PCa and its sensitivity to detect lesions in patients with clinically significant disease. The study involved 531 patients (ClinicalTrials.gov Identifier: NCT02615067). Results revealed that [^99m^Tc]Tc-MIP-1404 was able to visualize PCa lesions with specificity in the range of 71–75%, but failed to achieve a sufficient level of sensitivity as formulated in the co-primary endpoint [17].

The second PSMA agent labeled with technetium-99m was [^99m^Tc]Tc-PSMA-I&S developed as an alternative to [^111^In]In-PSMA-I&T, a useful agent for localizing small PCa metastases and performing radio-guided surgery (RGS) [115]. First-in-human administration of [^99m^Tc]Tc-PSMA-I&S in a patient with metastatic castration-resistant PCa and one with histologically confirmed primary PCa showed high plasma protein binding of the tracer (94%), which promoted its efficient uptake in PCa lesions over time, with a steady increase in lesion-to-background ratios up to 21 h after injection [115]. Moreover, preoperative SPECT/CT imaging demonstrated that [^99m^Tc]Tc-PSMA-I&S was highly accumulated in all lesions previously identified by [^68^Ga]Ga-PSMA-11 [115]. Maurer et. al. demonstrated the high value of [^99m^Tc]Tc-PSMA-I&S in intraoperative detection of recurrent PCa in 31 patients with four or less metastatic soft tissue lesions previously determined by [^68^Ga]Ga-PSMA-11. The authors found that [^99m^Tc]Tc-PSMA-I&S correctly identified and facilitated surgical removal of metastasis in a range of size from 3 to 25 mm and was able to detect additional metastases in two patients who were not detected with preoperative [^68^Ga]Ga-PSMA-11. However, due to the inherent limitations of SPECT/CT imaging, [^99m^Tc]Tc-PSMA-I&S was able to detect only 25 of the 44 lesions (56.8%) observed with [^68^Ga]Ga-PSMA-11 PET imaging [137]. Recently, Mix et al. proved that RGS allowed distinguishing between tumor-bearing and tumor-free LNs by means of a simple ex situ analysis of [^99m^Tc]Tc-PSMA-I&S uptake in LN [138]. However, Werner et al. pointed out that detection rates in patients with biochemical recurrence and low PSA levels (<4 ng/mL), obtained by [^99m^Tc]Tc-PSMA-I&S imaging at various clinical stages, were clearly lower than previously reported for [^68^Ga]Ga-PSMA-11 PET-imaging [139]. Consequently, the authors concluded that [^99m^Tc]Tc-PSMA-I&S could be used for primary staging and for restaging of advanced recurrent PCa only if PSA levels are greater than 4 ng/mL. Albalooshi et al. found similar results in a comparative study including 28 PCa patients, in which [^68^Ga]Ga-PSMA-11 imaging outperformed over [^99m^Tc]Tc-PSMA-I&S’ since the first permitted the detection of lesions in 25 patients (89.2%) while the second detected lesions only in 20 patients (71.4%) of. No patients with positive [^99m^Tc]Tc-PSMA-I&S SPECT/CT had negative [^68^Ga]Ga-PSMA-11 PET/CT. Nevertheless, no patient with PSA levels greater than 2.1 ng/mL presented discordant results between the two sets of images and the detection of LNs and BM was not significantly different [140].

Another [^99m^Tc]Tc-PSMA RP under clinic investigation is [^99m^Tc]Tc-EDDA/HYNIC-Lys(Nal)Urea-Glu (here referred to as [^99m^Tc]Tc-EDDA/HYNIC-PSMA). Ferro-Flores et al. carried out the first in-human studies in three healthy men to determine the [^99m^Tc]Tc-EDDA/HYNIC-PSMA pharmacokinetics; the results showed that the complex presented rapid blood clearance with renal elimination and was able to detect tumors and metastases of PCa with high sensitivity, similar to. [^68^Ga]Ga-PSMA-617 [117]. Preliminary studies in eight patients with histologically confirmed PCa demonstrated the ability of [^99m^Tc]Tc-EDDA/HYNIC-PSMA to specifically recognize PCa tumors and their metastases with an average tumor/background ratio of 8.99 ± 3.27 at 3 h [141]. A direct comparison of [^99m^Tc]Tc-EDDA/HYNIC-PSMA with [^68^Ga]Ga-PSMA-11 in 14 patients with PCa, demonstrated the good diagnostic sensitivity of [^99m^Tc]Tc-EDDA/HYNIC-PSMA imaging, which was able to detect 100% of prostate lesions, 91.7% of bone lesions and all LN metastases greater than 10 mm. However, its sensitivity was lower than that of [^68^Ga]Ga-PSMA-11 PET/CT imaging, when the LNs lesions were smaller than 10 mm, since in this case only 28% of the lesions were detected by [^99m^Tc]Tc-EDDA/HYNIC-PSMA [142]. Consequently, its use was not recommended in patients with small-volume lesions. However, Garcia-Perez et al. demonstrated that after a qualitative and semiquantitative comparison in 23 patients with PCa metastases in the prostate, bone, and LNs, with dimensions between 5-21 mm, the [^99m^Tc]Tc-EDDA/HYNIC-PSMA and [^68^Ga]Ga-PSMA-11 images were comparable [143].

[^99m^Tc]Tc-EDDA/HYNIC-PSMA scan was also compared with [^99m^Tc]Tc-MDP scan in 41 patients with histologically confirmed PCa to assess the sensibility for BM detection. The results of this preliminary study did not show significant differences between ^99m^Tc-RPs in the detection of BM and demonstrated an additional benefit of [^99m^Tc]Tc-EDDA/HYNIC-PSMA, which provided additional information by detecting residual disease in two patients and lymph nodal metastases in seven patients [144].

Recently, Vallejo-Armenta et al. studied [^99m^Tc]Tc-EDDA/HYNIC-PSMA SPECT imaging in 41 patients with suspected brain tumors, to diagnose tumoral neovasculature proliferation in brain metastases and gliomas. [^99m^Tc]Tc-EDDA/HYNIC-PSMA SPECT images showed low concentrations of RP in low-grade gliomas due to the minimal expression of PSMA in this tissue. In contrast, increased uptake was found in brain metastases, with recurrent and high-grade gliomas due to PSMA overexpression in the proliferating microvasculature of the brain and the vascular endothelium of grade IV gliomas. Based on these results, the authors stated that [^99m^Tc]Tc-EDDA/HYNIC-PSMA can also be considered a potential neuroimaging agent to assess tumoral neovasculature formation in gliomas and brain metastases [145].

Fallahi et al. compared [^99m^Tc]Tc-EDDA/HYNIC-PSMA versus [^68^Ga]Ga-PSMA-11 in the evaluation of 22 patients with metastatic PCa [146]. Both agents were able to detect the lesions in 21 of 22 patients, showing absolute agreement and a significant correlation between serum PSA level and the imaging of the disease detected by the two modalities, although the number of lesions detected in the prostate gland bed was significantly different between the modalities. Fallahi et al. explained that these differences could be due to more scatters from the bladder and lower spatial resolution of SPECT compared with PET imaging. However, they found that [^99m^Tc]Tc-EDDA/HYNIC-PSMA delayed imaging leads to a better target-to-background ratio, which may obviate its limitation in the detection of prostate bed lesions [146].

[^99m^Tc]Tc-HYNIC-ALUG, has been evaluated to improve the diagnosis and follow-up of therapy in PCa patients [109]. Liu et al. demonstrated the effect of PSA concentration on tumor detection by ^99m^Tc-HYNIC-ALUG SPECT/CT imaging in patients with radical prostatectomy and biochemical recurrence. The calculated overall positivity rate of [^99m^Tc]Tc-HYNIC-ALUG PSMA SPECT/CT turned out to be 72.6%, and for efficient detection of metastasis, the PSA level must be greater than 1.30 ng/mL [147]. This PSA value is almost 10 times higher than that reported by Schmidkonz et al. using [^99m^Tc]Tc-MIP-1404 [136]. However, the positive detection rate was as low as 49% for a PSA level between 0.2 and 0.5 ng/mL and increased to 89% for a PSA level between 3 and 5 ng/mL. Performing a retrospective study on PCa patients with biochemical recurrence, Su et al. found that SPECT/CT imaging with [^99m^Tc]Tc-HYNIC-ALUG can identify more metastatic lesions and offers a higher detection rate than other imaging techniques such as MRI and bone scanning, even when the patient PSA levels are low [148]. Recently, this agent has also been investigated in image-guided surgery [33] and to predict the early response to treatment with carbon ion radiotherapy in PCa [149], showing positive results in both cases.

The last PSMA derivative under clinical investigation is [^99m^Tc]Tc-PSMA-T4 a new RP developed at the National Center for Nuclear Research Radioisotopes Centre POLATOM [111]. The diagnostic utility of this RP has been very recently reported by Cwikla and co-workers [150]. In this pilot study, the authors assessed the clinical value of [^99m^Tc]Tc-PSMA-T4 images in terms of tumor extent in patients with confirmed PCa that qualified them for initial therapy and tumor recurrence evaluation with promising results. Data from the analysis of sensitivity/specificity were: 92%/100% for primary tumors; 83%/100% for pelvic LN; 100%/95% for other LNs and soft tissues, and for BM were both 100%. In another investigation, Sergieva et al. also evaluated the clinical application of ^99m^Tc-PSMA-T4 SPECT-CT imaging in 36 patients with recurrent PCa, and PSA ranging from 73 to 0.12 ng/mL [151]. Results reported 84,37% of sensitivity, 100% of specificity, and 86,11% of accuracy. The authors suggested that [^99m^Tc]Tc-PSMA-T4 could be used for the diagnosis of recurrent disease to determine the personalization of treatment in patients with PCa and biochemical progression if PSA is higher than 0.5 ng/mL [151].

Singh and coworkers performed a head-to-head comparison between [^68^Ga]Ga-PSMA-11 PET/CT and [^99m^Tc]Tc-PSMA-T4 whole-body and regional SPECT/CT for the detection of metastases in 10 patients with metastatic PCa [152]. The authors reported that [^68^Ga]Ga-PSMA-11 whole-body PET imaging reconstructed in 3D was useful in detecting 112 lesions in all 10 patients, while ^99m^Tc-PSMA-T4 planar whole-body (anterior and posterior) images detected only 57 lesions (51%) in 9 of the 10 patients. However, when regional ^99m^Tc-PSMA-T4 SPECT/CT imaging was used, the detection rate increased to 61.0%.

## 5. Multimodal and Multipurpose Systems as the Ultimate Perspective in [^99m^Tc]Tc-Housed PSMA-i

Considering the perspectives for this area of improvement, we stress that the high chemical versatility of Glu-Ureo-motif can also support the development of multivalent/multireceptor agents to increase the tumor uptake and bypass the tumor heterogeneity [153], as well the generation of hybrid agents for multipurpose strategies [154]. The latter can be designed to combine the good nuclear properties of a [^99m^Tc]Tc gamma-probe with fluorescent groups useful for fluorescent imaging in a bimodal approach for preoperative, intraoperative, and postoperative imaging, or different functional groups/chelators for theranostic applications thus improving the prognosis of the treatment and therapeutic efficacy along with the best safety profile [155,156].

**Figure 12:**
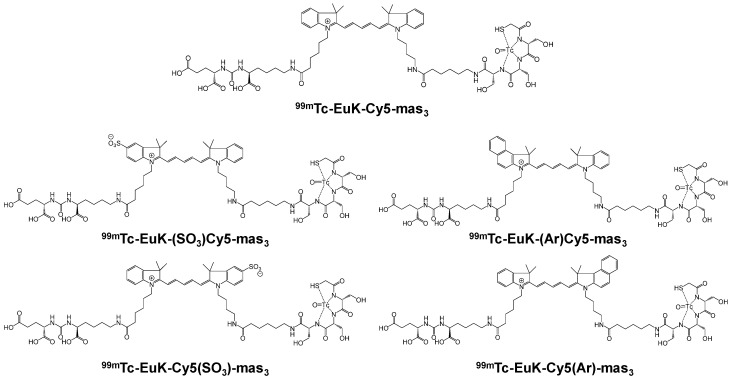
Schematic drawing of bimodal hybrid tracer based on fluorescent Cy5 dye.

In this regard, very recently Hensbergen and coworkers developed six [^99m^Tc]Tc-labeled Glu-Urea-based agents, incorporating cyanine backbones as an optical marker, for targeting PSMA in image-guided surgical interventions [157] with the benefit of being augmented with intraoperative optical identification of the lesion. Compounds were designed by inserting differently substituted Cy5 dye between Glu-Urea- targeting moiety and mas_3_ chelator to study the dye’s interaction with the amphipathic entrance funnel of the receptor (Figure 12).

Bimodal compounds were assessed for their chemical and photophysical properties and PSMA affinity. In vivo SPECT-imaging, biodistribution and fluorescence imaging were performed on BALB/C mice with orthotopically transplanted PC346C tumors.

The authors found that changes in dye composition impacted the photophysical properties, stability in serum (range 76% ± 0–89% ± 6%), plasma protein interactions (range 85.0% ± 2.3–90.7% ± 1.3%), PSMA affinity (IC50 range 19.2 ± 5.8–175.3 ± 61.1 nM) and in vivo features (tumor-to-prostate and tumor-to-muscle ratios range 0.02 ± 0.00–154.73 ± 28.48 and 0.46 ± 0.28–5157.50 ± 949.17, respectively; renal, splenic, and salivary retention).

Interestingly, the introduction of an anionic sulfonate group on the PSMA-i-bearing agent increased the tracer PSMA affinity and influenced the in vivo performance [157].

Bimodal radiohybrid conjugates containing radionuclides for imaging with the possibility of therapeutic radionuclides usage have been recently investigated at the Technical University of Munich as a new paradigm for the development of theranostic compounds [155].

This new category of theranostic agents includes PSMA-i conjugates that can transfer two different radionuclides due to the concurrent presence of distinct functional groups/chelators, one for a diagnostic radionuclide the other for radiotherapy. These compounds are named radio-hybrid PSMA-i agents. They combine ^18^F or ^68^Ga for PET imaging with o ^177^Lu or ^225^Ac -chelate for therapy. PSMA-i ligands were designed to carry in one molecule a silicon-fluoride-acceptor for ^18^F isotopic exchange and a DOTA-based chelator to accommodate the radio-metal, assuring fast, efficient, and site-specific radiolabeling. The synthesis was performed using a mixed solid-liquid-phase synthesis strategy [155,156]. In this connection, technetium might be very useful for the development of SPECT/radiotherapy hybrid agents: indeed, the chelating systems described above are highly selective for this metal, guaranteeing efficient and site-specific radiolabeling and negligible transmetalation. These features may open the door to new perspectives for a novel category of [^99m^Tc]Tc-RPs.

## 6. Conclusions

Although the literature describes different [^99m^Tc]Tc-housed PSMA-i agents and some of them are under clinical investigation, beyond the intrinsic superiority of PET over SPECT technology, none outperforms PET agents. Thus, [^99m^Tc]Tc-PSMA-is are in general recommended when [^68^Ga]Ga-PSMA-is are not available. Looking at the plethora of PSMA-i RPs proposed over recent years for various radiopharmaceutical applications, it is evident that the development of these agents has been simplified by the high versatility of the Glu-Ureo-motif to accept diverse and even bulky chemical modifications, without losing PSMA targeting efficiency. Such high plasticity toward modification, joined with the reach and versatile coordination chemistry of technetium and the availability of various chemical approaches for selective and efficient [^99m^Tc]Tc-chelation, could support the development of chemical strategies for the optimization of the biological profile of the radiolabeled compounds, as well as the fine-tuning of their pharmacokinetic profiles, thus improving their diagnostic value and offering a unique opportunity to find a solution to produce a more effective counterpart of [^68^Ga]Ga-RPs.

## Figures and Tables

**Table 1:** Pros and cons of the use of Technetium-99m radionuclide vs. Gallium-68 and Indium-111 radionuclides.

^99m^Tc (SPECT)	^68^Ga (PET)	^111^In (SPECT)
**PROS**. Optimal nuclear properties (t_1/2_ = 6.02 h; Eγ = 140 keV). t_1/2_ is sufficiently long, allowing completing RP synthesis in radiopharmacies, distribution to hospitals, dose preparation, administration, and collection of clinically useful images. The monochromatic 140 keV photons (90% abundance) are ideal for obtaining SPECT imaging with high spatial resolution using commercial gamma cameras. Low radiation exposure to patients and to personnel due to the absence of corpuscular radiation. ^99m^Tc half-life nicely matched the PK of small and medium-size peptides. On-site availability from inexpensive portable 9^9^Mo/^99m^Tc generator system, which overcomes the problem of worldwide distribution. Rich and versatile coordination chemistry. ^99m^Tc-RPs are efficiently prepared in situ through commercially available lyophilized instant kits. ^99m^Tc-RPs produce lower radiation effective doses than ^68^Ga-RP. Wider distribution of SPECT technology over PET. **CONS**. SPECT has lower spatial resolution (7–8 mm) than PET. The sophisticated Tc chemistry might complicate the setting of instant cold kits.	**PROS**. Good nuclear properties (t_1/2_ = 67.71 min; E_β__+_ = 1899 keV). t_1/2_ is sufficiently long to allow the synthesis, purification, dose preparation of RP, administration and collection of clinically useful images. ^68^Ga t_1/2_nicely matched the PK of peptides On-site availability from portable 6^8^Ge/^68^Ga-generator system. The long t_1/2_ of ^68^Ge (270.8 days) allows the utilization of a single generator for a long period (1/2 y). ^68^Ga RPs can be produced through the use of kits that consent to a wide availability of the corresponding imaging agents. PET higher image resolution (4–5 mm) and relevant features over SPECT. **CONS**. The low t_1/2_ does not allow for widespread shipping of the isotope. The amount of ^68^Ga obtainable from generator in a single elution is sufficient to synthetize the tracer for only one or two patients, limiting the number of investigations for a single day. The higher energy of ^68^Ga positron with respect to ^18^F, can lead to lower spatial resolution and high radiation dose to the patients. The are fewer PET cameras installed worldwide with respect to SPECT ones. This limits the utility of this modality in daily clinical practice.	**PROS**. The long t_1/2_ (2.8 d) makes this radionuclide useful for SPECT imaging of biomolecules with slow kinetics, such as antibodies and their fragments. **CONS**. Suboptimal nuclear properties (t_1/2_ = 2.8 d; Eγ = 171 and 245 keV with 91% and 94% abundance, respectively). The high energy both γ rays produce low spatial resolution using commercial gamma cameras. Restrict availability of ^111^InCl_3_ due to the cyclotron production. High production costs.

**Table 2:** Summary of features of Technetium-99m labeling approaches.

	Tc(V) Oxo/Dioxo Core 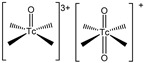	Tc(V)-HYNIC 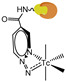	Tc(I)-Tricarbonyl 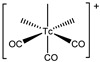	[Tc(V)N(PNP)]^2+^ 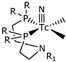
**Coordinating System (denticity; donor atoms set)**	Tetradentate; N_2_S_2_ N_3_S, N_4_	EDDA/Tricine; N_2_O_2_	Bi- or Tridentate; His-based ligands, polyamino-polycarboxylate, polyamines, aromatic and aliphatic amines, cyclopentadienyl ligands	Bidentate; Cys-based ligands [H_2_NS]^−^, [OS]^2−^
**Coupling BFC-molecular vector**	Difficult, potentially stressful for the molecular vector. Tetradentate ligand utilizes a carboxylic acid side-group to link biomolecules. Such ligands can be conjugated via carboxylic acids (COOH), amino (NH2), and thiocyanate (NCS) groups to biomolecules.	Easy, bioconjugate chemistry. HYNIC- conjugates are produced via an amide linkage formed by the reaction of the active ester derivative of the ligand.	Easy bioconjugate chemistry. The His-based chelator can be readily incorporated into the peptide chain by using traditional automated synthesis (peptides) or His-tag (proteins).	Easy, bioconjugate chemistry. The Cys-chelator can be readily incorporated into any peptide using standard solid- or solution-phase coupling methodologies; for proteins, native Cys-residues can be used or additional small Cys-terminal peptides can be attached even site-specifically.
**Labeling**	One-step	Two-step	One or Two-step	One or two-step
**Labeling Temperature**	Heating (75–100 °C)	Heating (60–100 °C) or RT	Generally heating (75–100 °C).	Heating (80–100 °C) or RT
**Purification**	No, very difficult if necessary	No, easy if needed	No, easy if needed	No, easy if needed
**Chemical identification**	Yes	No, difficult	Yes	Yes
**Isomerism**	Yes, two isomers (syn, anti) separable and individually evaluable.	Yes, several isomeric forms, not separable and not individually evaluable.	Potentially yes.	Yes, two isomers (syn, anti) separable and individually evaluable, having the same affinity for the target.
**Complex Stability**	Generally stable	Stable	Very stable	Very stable
**Flexibility against chemical-physical modulation**	Low, minimal variation of chelator backbone or by modification/insertion of PKM	Medium by chemical modification of Tricine and EDDA or by insertion or modification of a PKM	Medium by chemical variation of the chelator and modification/insertion of PKM	High by chemical modification of the substituent on PNP, variation of chelator and modification/insertion of PKM
**Kit formulation**	One vial	One vial	One/two vials	Two vials
**Feasibility for ^188^Re**	Yes	No	Yes	Yes

**Table 3:** PSMA-i labeled with [^99m^Tc][Tc(CO)_3_]^+^-fragment.

	Chelator Linker Scaffold	Affinity	Performance	Ref.
**[Tc(CO)_3_]1–7** **(2008)**	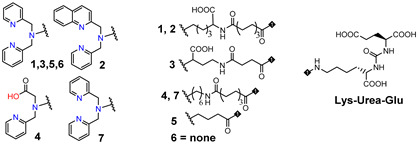	^a^ 10.75 (1) 0.50 (2) 10.34 (3) 0.17 (4) 0.91 (5) 199.56 (6) 2.06 (7)	Complexes were evaluated on PC3-PIP/PC3-flu xenografts. PSMA-dependent tumor uptake picked at 0.5–1 h *p.i*, followed by rapid washout. Rapid clearance from the kidneys. High spleen uptake followed by elimination. **Clinical translation:** No	[105]
**[Tc(CO)_3_]8–10** **(2013)**	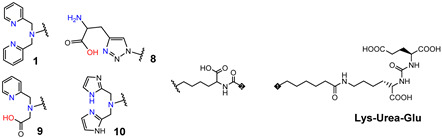	^b^ 15.25 1.12 (8) 12.60 (9) 16.30 (10)	Complexes were evaluated on PC3-PIP/PC3-flu xenografts. [Tc(CO)_3_]**8** is the best of the series. High and stable PSMA-dependent tumor uptake picked at 0.5 h *p.i*, uptake (28.31 ± 4.4% ID/g at 0.5 h vs. 23.22 ± 6.02% ID/g at 5 h *p.i*). High kidney uptake followed by slow washout. High spleen uptake followed by elimination. **Clinical translation:** No	[106]
**[Tc(CO)_3_]11–14** **(2013)**	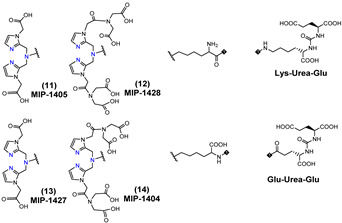	^c^ 4.35 ± 0.35 (11) 1.75 ± 0.32 (12) 0.64 ± 0.46 (13) 1.07 ± 0.89 (14)	Complexes were investigated on LNCaP/PC3 xenografts. High and stable tumor uptake ranged from 9.3 to 12.4% ID/g at 1 h *p.i* and 7.2–11.0% ID/g at 4 h. High tumor-to-blood ratios ranging from 29:1 to 550:1 and tumor–to–muscle ratios ranging from 31:1 to 157:1 at 4 h. Liver and intestinal uptake were <1% ID/g for all compounds. Rapid clearance from the kidneys. Low spleen uptake. **Clinical translation:** Yes	[34,107,108]

^a^ PSMA inhibitory affinity was determined using the corresponding rhenium complexes. Data are reported as *K_i_ (nM); ^b^ PSMA inhibitory affinity was determined by using the ligands, and data are reported as K_i_ (nM). ^c^ Binding specificity of ^99m^Tc-labeled PSMA-i was determined by saturation binding analysis in the presence of the corresponding rhenium compound or 2-PMPA. Data are reported as *K*d (nM).

**Table 7:** Formulation, indication, advantages and disadvantages of [^99m^Tc]Tc-PSMA RPs under clinical investigations.

**Agent**	**Formulation/Radiosynthesis**	**Indication**	**Pros/Cons**
**^99m^Tc-TrofolastatConcluded Phase 3** (**ClinicalTrials.gov Identifier: NCT02615067)**	Two-vial kit formulation, the composition and the procedure were not deeply described. Labeling: 1° step NaTcO_4_(40 mCi/1 mL saline) was added to the Isolink vial. Heat to 95 °C for 30 min. 2° Step: pH was adjusted to 7.5 with HCl.; PSMA-i (0.100 mg) was added. Heat to 95 °C for 30 min. Vol_tot_= n.d RCY 85%. Purification: by HPLC RCP ≥ 98%. Specific activity: 37 TBq/mmol.	Useful to assist in the initial diagnosis of PCa, the monitoring of disease progression and the response to the therapeutic treatment by SPECT imaging.	**Pros**. Lower urinary excretion, which allows for detecting PCa in the pelvic area at early stages of the disease. Successfully detected very small PCa lesions (<10 mm) in bone, LNs, and prostate gland, thus suggesting that SPECT/CT scan with this agent may provide prognostic information for both primary tumor and biochemical recurrence. Detection rates (50%) slightly lower than that of ^68^Ga-PSMA-11 at low PSA levels (0.2–1 ng/mL). **Cons**. Detection rates (50%) slightly lower than that of ^68^Ga-PSMA-11 at low PSA levels (0.2–1 ng/mL).
**^99m^** **Tc-PSMA-I&S** **Phase2 (ClinicalTrials.gov Identifier: NCT04832958)**	One vial: SnCl_2_, tartrate, PB pH 8, PSMA-I&S (35–40 µg). Labeling: NaTcO_4_ (20–30 mCi/1–5 mL), was added to the vial. pH 7.5–8, heat to 90 °C for 20 min. RCY 99%. Diluted to 10 mL. **A_m_**44–52 GBq/µmol.	Useful to localize small metastases of PCa and to perform RGS with the main advantage of the immediate confirmation and removal of metastasis prior of the histological analysis. Superior substitute of ^111^In PSMA-I&T.	**Pros**. Good visualization of the lesion also those previously identified with ^68^Ga-PSMA11. High accumulations in LNs metastases, allowing for the exact intraoperative identification ad resection during RGS. **Cons**. Lesion-to-background contrast is achieved after 5 h *p.i.* Detection rates in patients with biochemical recurrence and low PSA levels (<4 ng/mL), are lower than that reported for ^68^Ga-PSMA and for ^99m^Tc-Trofolastat.
**^99m^** **Tc-EDDA/HYNIC-PSMA Phase 1**	Two-vial kit formulation. 1 lyophilized vial: SnCl_2_ (0.020 mg), EDDA (20 mg), tricine (40.8 mg) mannitol (0.102 mg), HYNIC-PSMA-i (0.102 mg). 2° vial: Phosphate Buffer, 0.2 M pH 7. Labelling: to vial 1 was added PB (1 mL) and NaTcO_4_ (20–30 mCi/1.0 mL). Heat to 95 °C for 10 min. RCY ≥ 98%.	Useful to assist in the initial diagnosis of PCa and the monitoring of disease progression. by SPECT imaging. Suitable in planning RGS or for identification of PSMA+ lesions before consideration of radioligand therapy, e.g., with ^177^Lu-PSMA.	**Pros**. Faster blood clearance and urinary excretion, which allow the visualization, at 3 h *p.i.*, of PCa in the pelvic area at early stage of diseases. Lower liver uptake and lowest effective radiation dose than ^99m^Tc-Trofolastat. Able to localize the lesions in bone, soft tissues and LN. Lesion detection rate of 78.3% with respect to Ga-68 PSMA. **Cons**. Lower sensitivity than ^68^Ga-PSMA-11 for small-sized lesions. Only 28% of LNs lesions < 10 mm were detected, hence its use is not recommended in patients with small-volume disease.
**^99m^** **Tc-** **HYNIC-ALUG** **Phase 1**	HYNIC-ALUG (0.010 mg), EDDA (0.5 mL of 20 mg/mL in 0.1 M NaOH solution), Tricine (0.5 mL of 40 mg/mL in PBS 0.2 M, pH = 6.0). NaTcO_4_ (50 mCi/1.0 mL). SnCl_2_ (50 μL of 1 mg/mL in 0.1 M HCl solution). Heat to 100 °C for 15 min. Vol_tot_ = 2.050 mL RCY ≥ 98%.	Useful to assist in the initial diagnosis of PCa and the monitoring of disease progression by SPECT imaging. This is also proposed for RGS.	**Pros**. Efficient detection of the metastasis at PSA level greater than 1.30 ng/mL **Cons**. The high accumulation in the kidneys makes difficult the interpretation of scintigraphic images.
**^99m^** **Tc-** **PSMA-T4** **Phase 1**	Sterile and apyrogenic freeze-dry kit: PSMA-T4 (20 mg), Tricine (50 mg), EDDA (5 mg), SnCl_2_ (40 mg), Na_2_HPO_4_x12H_2_O (29 mg),/NaH_2_PO_4_x2H_2_O (3.0 mg). Labelling: NaTcO_4_ (20–30 mCi/1.0–2.5 mL). Heat to 95 °C, 10 min. RCY ≥ 95%.	Useful to assist in the initial diagnosis of PCa and the monitoring of disease progression. Suitable for RGS and for identification/assessment of PSMA^+^ lesions before consideration of radioligand therapy.	**Pros**. RP is efficiently prepared in a short time by freeze-drying PSMA-T4 kit. Favorable distribution and kinetic behavior which allow the visualization of the PSMA + lesions within 3 h *p.i*. **Cons**. The high accumulation in the kidneys and renal excretion can cause difficulties in images interpretation.
